# A Humanized Mouse Strain That Develops Spontaneously Immune-Mediated Diabetes

**DOI:** 10.3389/fimmu.2021.748679

**Published:** 2021-10-14

**Authors:** Sandrine Luce, Sophie Guinoiseau, Alexis Gadault, Franck Letourneur, Patrick Nitschke, Marc Bras, Michel Vidaud, Pierre Charneau, Etienne Larger, Maikel L. Colli, Decio L. Eizirik, François Lemonnier, Christian Boitard

**Affiliations:** ^1^ Laboratory Immunology of Diabetes, INSERMU1016, Department EMD, Cochin Institute, Paris, France; ^2^ Medical Faculty, Paris University, Paris, France; ^3^ Biochemistry and Molecular Genetics Department, Cochin Hospital, Paris, France; ^4^ Molecular Virology and Vaccinology, Pasteur Institute, Paris, France; ^5^ Diabetology Department, Cochin Hospital, Paris, France; ^6^ Université Libre de Bruxelles (ULB) Center for Diabetes Research, Medical Faculty, Université Libre de Bruxelles, Brussels, Belgium; ^7^ Diabetes Center, Indiana Biosciences Research Institute (IBRI), Indianapolis, IN, United States

**Keywords:** autoimmunity, type 1 diabetes (T1D), humanized mouse, HLA-DQ8, epitopes, preproinsulin

## Abstract

To circumvent the limitations of available preclinical models for the study of type 1 diabetes (T1D), we developed a new humanized model, the YES-RIP-hB7.1 mouse. This mouse is deficient of murine major histocompatibility complex class I and class II, the murine insulin genes, and expresses as transgenes the HLA-A*02:01 allele, the diabetes high-susceptibility HLA-DQ8A and B alleles, the human insulin gene, and the human co-stimulatory molecule B7.1 in insulin-secreting cells. It develops spontaneous T1D along with CD4^+^ and CD8^+^ T-cell responses to human preproinsulin epitopes. Most of the responses identified in these mice were validated in T1D patients. This model is amenable to characterization of hPPI-specific epitopes involved in T1D and to the identification of factors that may trigger autoimmune response to insulin-secreting cells in human T1D. It will allow evaluating peptide-based immunotherapy that may directly apply to T1D in human and complete preclinical model availability to address the issue of clinical heterogeneity of human disease.

## Introduction

Type 1 diabetes (T1D) is a multifactorial autoimmune disease that remains a major health challenge (1). Its incidence increases by 3% to 4% yearly. There is presently no therapy to definitively revert or stop the autoimmune process responsible for the destruction of β cells (2). Upstream of therapies, immunological markers for the autoimmune response to β cells have shown limitations in predicting the development of type 1 diabetes in subjects with prediabetes, especially when a single autoantibody is detected (3). Following their use in preclinical models of T1D, antigen and peptide-specific immunotherapies have been proposed as strategies with a low risk/benefit ratio in human. Early attempts have shown minimal efficacy in human, as in using glutamic acid decarboxylase-65 (GAD65) (4). However, the use of an immunodominant proinsulin peptide has proven to be well-tolerated and to delay C-peptide decline in human (5). From both a diagnostic and a therapeutic standpoint, preclinical models of T1D have fallen short of translating into human. Current models do not allow testing peptides derived from human autoantigens that may directly apply to the human situation in vaccination strategies. A new preclinical model to study T1D in a humanized mouse model would be amenable to evaluate the relevance of T-cell assays or peptide immunotherapy that would directly apply to human diabetes.

Our aim in this study was to create a preclinical model that would develop spontaneous T1D and allow characterizing HLA-A class I and class II MHC-restricted peptides that directly apply to human. We chose human preproinsulin (hPPI) as a major T1D autoantigen (6–8), the most common class I MHC HLA-A*02:01 allele in the three major ethnic groups (50% in Caucasian and Asian and 30% in African) (9) and the high T1D susceptibility class II DQ8 A and B alleles (10). We previously generated the YES mouse that expresses the HLA-A*02:01, the *HLA-DQ8*, and the *human insulin* (*hINS*) genes and fails to develop spontaneous T1D, but develops T1D when challenged with polyinosonic-polycytidylic acid (pI:C) (11). As the expression of B7.1 in pancreatic β cells has been shown to trigger the development of T1D in conventional mice (12) and accelerate diabetes in the NOD mouse (13), we introduced the human costimulatory molecule B7.1 (*hB7.1*) under the control of the rat insulin promoter in pancreatic β cells onto the YES background to enforce the development of spontaneous T1D. The first objective was to identify autoreactive epitopes from hPPI involved in the T1D autoimmune response and evaluate their relevance in human. The second objective was to identify external factors that may accelerate the development of T1D within the frame of human MHC presenting molecules (14).

This new humanized mouse expressing HLA-A*02:01, *HLA-DQ8*, *hINS*, and the *RIP-hB7.1* transgenes, thereafter called YES-RIP-hB7.1 mice, develops spontaneous T1D and shares immunological features with human T1D. This new model of spontaneous diabetes completes the previously reported YES model. According to the clinical complexity of human T1D that is likely to be a heterogeneous set of diseases, these new models altogether provide a larger set of preclinical tools to study human T1D.

## Materials and Methods

### Patients

Patients (**Table 1**) were recruited under a human protocol of clinical trial for the access of T1D and healthy donor peripheral blood mononuclear cells (PBMCs) in agreement with the Research Ministry Authorization (MESR number DC-2015-2536/IDRCB number 2015-A01875-44). Recent-onset T1D patients (18 males, 12 females, aged 35.3 ± 17.5 years) were studied within 3 months from diagnosis (9.4 ± 14.5 days, range 1–75 days). HLA genotyping was performed by AmbiSolv genotyping (Dynal/Invitrogen) on DNA with Gentra Puregene Blood kit A (Qiagen). Eleven recent-onset T1D patients expressed HLA-DQ8 (DQA*03:01, DQB*03:02) and 19 co-expressed HLA-DQ8 and HLA-DQ2 (DQA*05:01, DQB*02:01). Long-standing HLA-DQ8-expressing T1D patients (3 males, 3 females, ages 55.25 ± 21.93 years) had a median of diabetes duration of 5.34 ± 2.6 years (range 0.7 to 7 years). Control subjects were healthy donor (*n* = 9, 3 males, 6 females, aged 34 ± 18.6 years) and T2D controls (*n* = 4, 1 male, 3 females, aged 67.7 ± 4.57 years) and only express HLA-DQ8 molecule.

**Table 1:** Patients and healthy donors.

T1D patient and controls	Gender	Age (years)	Diabetes duration	Autoantibodies	HLA genotyping
R1	F	61	4 days	GAD:158-IA2:52.7-ZnT8:915	DQ8
R2	F	27	30 days	GAD:13-IA2:neg-ZnT8:1324	DQ2/DQ8
R3	M	44	7 days	GAD:neg-IA2:13ZnT8:neg	DQ8
R4	F	27	1 day	GAD:neg-IA2:28-ZnT8:324	DQ2/DQ8
R5	M	19	1 day	GAD:28-IA2:neg-ZnT8:300	DQ2/DQ8
R6	F	25	4 days	GAD:18-IA2:31-ZnT8:1001	DQ2/DQ8
R7	M	36	2 days	GAD: 500-IA2:neg-778	DQ2/DQ8
R8	M	27	2 days	GAD:1500-IA2:28.9	DQ2/DQ8
R9	F	19	3 days	GAD:neg-IA2:neg-ZnT8:2081	DQ2/DQ8
R10	M	48	28 days	GAD:20.8-IA2:35-ZnT8:1086	DQ8
R11	F	35	56 days	GAD:190-IA2:47-ZnT8:neg	DQ2/DQ8
R12	M	26	1 day	GAD:12-IA2:neg-ZnT8:151	DQ2/DQ8
R13	M	30	1 day	GAD:9-IA2:neg-ZnT8:32	DQ2/DQ8
R14	M	28	3 days	GAD:64-IA2:32-ZnT8:neg	DQ2/DQ8
R15	M	19	1 day	GAD:28-IA2:22-ZnT8:neg	DQ2/DQ8
R16	M	24	30 days	GAD:21-IA2:37	DQ8
R17	F	69	5 days	GAD:25-IA2:39-ZnT8:1665	DQ2/DQ8
R18	M	79	10 days	GAD:127-IA2:1.4-ZnT8:neg	DQ2/DQ8
R19	F	36	28 days	GAD:46-IA2:232-ZnT8:neg	DQ8
R20	M	28	2 days	GAD:400-IA2:neg-ZnT8:388	DQ2/DQ8
R21	M	19	8 days	GAD:50-IA2:125-ZnT8:neg	DQ8
R22	M	73	3 days	GAD>2000-IA2:86-ZnT8:466	DQ2/DQ8
R23	F	17	7 days	GAD>2000-IA2:neg-ZnT8:neg	DQ8
R24	M	33	3 days	GAD>2000-IA2>4000-ZnT8:800	DQ2/DQ8
R25	F	24	6 days	GAD:448-IA2:neg-ZnT8:neg	DQ2/DQ8
R26	M	19	1 day	GAD>2000-IA2:3141-ZnT8:156	DQ8
R27	M	26	4 days	GAD>2000-IA2:91-ZnT8:neg	DQ8
R38	F	58	75 days	GAD>2000-IA2>4000-ZnT8:884	DQ8
R29	M	28	2 days	GAD:neg-IA2:neg-ZnT8:neg	DQ8
R30	F	55	2 days	GAD>2000-IA2:neg-ZnT8:neg	DQ2/DQ8
L1	F	41	7 years	GAD:222-IA2 neg-ZnT8 neg	DQ8
L2	M	55	7 years	GAD:20-IA2 neg	DQ8
L3	F	86	6 years	GAD:122-IA2 neg-ZnT8 neg	DQ8
L4	F	46	6 years	GAD:10-IA2 neg	DQ8
L5	M	35	0.7 years	GAD:190-IA2:47-ZnT8 neg	DQ8
L6	M	54	0.7 years	GAD-neg-IA2 neg-ZnT8:18.6	DQ8
H1	M	55	nd		DQ8
H2	F	32	nd		DQ8
H3	M	59	nd		DQ8
H4	H	24	nd		DQ8
H5	F	44	nd		DQ8
H6	F	36	nd		DQ8
H7	M	21	nd		DQ8
H8	M	74	nd		DQ8
H9	F	28	nd		DQ8
T2D1	F	69	24	neg	DQ8
T2D2	F	61	23	neg	DQ8
T2D3	F	70	19	neg	DQ8
T2D4	M	71	14	neg	DQ8

R, recent-onset T1D patients; L, long-standing T1D patients; H, healthy control subjects; T2D, type 2 diabetic control; neg, negative result; nd, non-diabetic.

### Mice

YES-RIP-hB7.1 mice were obtained by lentiviral transgenesis of YES mice (*H-2 D^b^*, *mouse β2 microglobulin*, *IAα^b^*, *β^b^*, *IEβ^b^* quintuple KO mice expressing a chimeric-α3 H-2 D^b^ domain, human β2 microglobulin-HLA-A*02:01 monochain molecule named HHD- and the HLA-DQB1*03.02 and HLA-DQA1*03.01 genes) (15, 16). The *RIP-hB7.1* transgene was inserted in a HIV-derived recombinant lentiviral vector (LV-RIP-hB7.1; insert of 4,640 bp) as previously reported into YES mice (17, 18). Retroviral pseudotypes were injected into fertilized eggs obtained from super-ovulated female YES mice mated with male YES mice (16). Fertile and transduced eggs were reimplanted into pseudo-pregnant C57BL/6xCBA F1 mice. All mice were maintained under specific pathogen-free conditions, and experimental studies were performed in accordance with the Institutional Animal Care and Use Guidelines and accredited by the Ethics Committee n°34 of Paris Descartes under number CEEA34.CB.024.11. Mice were monitored three times a week for glycosuria. When glycosuria was detected, diabetes was diagnosed when two successive glycemic values >250 mg/dl were detected at 24 h interval. Diabetes incidences curves correspond to the percentage of mice diagnosed as diabetic referring to the aforementioned criteria.

### Molecular Characterization of LV-RIP-hB7.1 Transgene

YES mice submitted to lentiviral transgenesis were genotyped using the following primers: 5′end (AGGGAACATCACCATCCAAG) and 3′end (TGCCAGTAGATGCGAGTTTG), annealing temperature: 62°C, amplicon: 181 bp. A positive RIP-hB7.1 mouse was selected to perform the molecular characterization of the *LV-RIP-hB7.1* transgene. To characterize insertion sites in genomic DNA of the founder, we realized a sequence capture design using the SeqCap EZ system (Roche NimbleGen) targeting 97.6% of the transgene. After nebulization, fragmented genomic DNA was end-repaired and ligated with adapters. A double capture was realized to obtain a GS Junior library ready for emulsion PCR (emPCR). Fragments were then annealed to capture beads and clonally amplified by emPCR (emPCR Amplification Method Manual LibL; GS Junior Titanium Series, Roche). Beads with the cloned amplicons were then enriched, loaded on a 454 picotiter plate, and sequenced on the Roche GS Junior Sequencer according to the protocol of the manufacturer (Sequencing Method Manual, GS Junior Titanium Series, Roche). Image analysis and base calling of the raw sequencing data were performed using the default “shotgun” Roche GS Junior data analysis pipeline. To obtain the position of transgene insertions in genomic DNA, sequence reads were aligned to the 50 bp of each end of the transgene primary sequence using BWASW, an algorithm designed for long reads with more errors. Aligned sequences were then aligned against C57BL/6NJ genomic sequences to obtain scaffold at both ends of the transgene sequence. A 10 kb for each region was extracted and amplified by long range PCR (LR-PCR). A nested PCR targeting the *hB7.1* transgene was performed on the long range PCR product to highlight the presence of the transgene and identify the sequence of the insertion site of the LV-RIP-hB7.1 transgene on genomic DNA. A genotyping PCR was designed from the region overlapping genomic DNA and LV-RIP-hB7.1 transgene using the following primers: 5′end (TAAATGCAGGGCTCCAGACT) and 3′end (TAGTGTGTGCCCGTCTGTTG), annealing temperature: 62°C, amplicon: 645 bp.

To compare the genetic background of YES-RIP-hB7.1 and YES mice, we performed a GenScan SNP Affymetrix. Briefly, high-quality genomic DNAs (250 ng) were digested with *Nsp*I and *Sty*I enzymes. *Nsp*I and *Sty*I adaptors were then ligated to restricted fragments followed by PCR using the universal primer PCR002. Each amplicon was purified, pooled, and used for fragmentation and end-labeling with biotin using terminal transferase. Labeled targets were then hybridized overnight to Genechip^®^ Mouse Diversity Genotyping Array (Affymetrix, ref: 901615) at 49°C. Chips were washed on the fluidic station FS450 following specific Affymetrix protocols, scanned with the GCS3000 7G, and analyzed using GCOS software to obtain raw data (.cel files). Genotypes were identified by the Affymetrix Genotyping Console tool using Dynamic Model (DM) and Bayesian Robust Linear Model with Mahalanobis (BRLMM) mapping algorithms. Genotypes were then extracted for each SNP of the chip. Big data were exploited in R console software. We submitted the data generated by the Affymetrix SNP Array detection to a public repository (http://www.ncbi.nlm.nih.gov/geo) and the GEO Series accession numbers were GSE101551 and GSE151644.

### *In Vivo* T-Cell Depletion Treatment and pI:C Stimulation

For *in vivo* T-cell depletion, YES-RIP-hB7.1 mice were injected with four weekly injections (i.p.) of 1 mg of anti-CD4 (GK1.5 clone) (19) and 1 mg of anti-CD8 (YTS 169.4 clone) (20) starting at 2 or 8 weeks of age and monitored for diabetes development. For *in vivo* poly(I:C) stimulation, YES and YES-RIP-hB7.1 mice (8 weeks old of age) were submitted to seven daily injections (i.p.) of 100 µg of poly(I:C) VacciGrade (vac-pIC, Invivogen) and monitored for diabetes development (16).

### Cytotoxic Assay

T-cell cytotoxicity assay was performed on *HHD*-transfected P815 cells prepulsed with 10 μg peptide for 2 h at 37°C using the LDH Cytotoxicity Detection Kit^PLUS^ (Roche) or *hPPI/HHD*-doubled transfected P815 cells. High control of lysis corresponded to cell killing with a lysis solution (Tween-20), which provide the maximum LDH release. Low control of lysis, which provide the spontaneous cytotoxicity, was evaluated on cells that were not submitted to additional treatment. Specific lysis was calculated as optical density of (targeted condition − spontaneous lysis)/(maximum LDH release − spontaneous lysis) × 100.

### Enzyme-Linked Immunospot Assay

IFNγ-ELISpot assays were achieved as previously reported (21). Spots were counted using Bioreader 5000 Pro Sf (BioSys GmbH). Splenocytes from mice were stimulated overnight with peptides and Il-2 (5 U/ml final). The enzyme-linked immunospot (ELISpot) assay was performed using mouse γ-ELISpot antibody pair, from U-CyTech biosciences. Data are the mean of triplicate wells. The background of IFNγ responses was evaluated in the absence of peptide. Specific IFNγ responses were expressed as spot-forming cells (SFC) per 10^6^ cells after normalization of the background. Positive controls were triplicates of cells stimulated by 1 μg/ml ConA (Sigma-Aldrich) and negative controls by pyruvate dehydrogenase (PDHase_208-216_) irrelevant peptide.

### Human Recombinant-PPI Protein Production

The cDNA sequence of preproinsulin was mutated to convert the Ala codon in position 3 of the leader DNA sequence to Asp by site-directed mutagenesis (NEB) to abolish the signal-sequence site cleavage of PPI (22). Mutated PPI was cloned in pFastBac vector to generate the PPI-recombinant bacmid, then PPI-recombinant Baculovirus to produce human PPI protein into Bac-to-Bac Baculovirus Expression System (Invitrogen) according to the instruction of the manufacturer. Purification was performed on pelleted infected insect cells, following preparation and extraction procedures for insoluble proteins (inclusion body) from *Escherichia coli* (23). Purity was confirmed on SDS/PAGE gel and quantification was performed using the BCA Protein assay kit (Pierce).

### Cell Proliferation Assay

Spleen cells (10^5^/well) were incubated with 0.5 μg antigen/well or 0.1 µg peptide/well for 72 h at 37°C in triplicate. Proliferation was evaluated with BrdU Cell Proliferation Assay Kit (Cell Signaling) and expressed as proliferation index (PI). Background and positive controls were evaluated in triplicate wells containing 10^5^ cells/well incubated without antigen or in the presence of 10 μg/ml final concentration of anti-mouse CD3ϵ antibody, respectively.

### Immunohistochemistry and Fluorescence

Immunofluorescence stainings were performed on formalin-fixed paraffin pancreas sections that were deparaffinized in xylene and dehydrated by ethanol. After washing, antigen retrieval was realized by hot incubation in citrate buffer, followed by permeabilization (20 min in PBS 1×/0.4% Triton X-100) and saturation (30 min PBS 1×/1% horse serum) before immunostaining with biotinylated rat anti-human CD3ε(AbD Serotec) and polyclonal rabbit anti-glucagon (DAKO) antibodies overnight. Slides were washed with PBS 1×/1% BSA/0.1% Triton X-100 and stained with an anti-rabbit Ig-FITC antibody (Abcam) and SAV-Cy3 (Abcam) at RT. Sections were mounted in Vectashield mounting medium for fluorescence with DAPI (Vector Laboratories). Observations were made with a spinning disk confocal apparatus at the Cochin Institute Imaging Platform and pictures analyzed with the ImageJ software.

Stable immunohistochemical stainings were performed on paraffin-embedded pancreas sections and stained with polyclonal guinea-pig anti-human insulin (DAKO) or rat anti-mouse CD4 biotin (eBioscience), followed by incubation with peroxidase-labeled antibodies. All reactions were revealed with diaminobenzidine (DAB, Genemed). Sections were counterstained with hematoxylin and mounted. Observations were made with Zeiss AxioObserver Z1 microscope coupled with MRm Axiocam Zeiss and pictures analyzed with the ImageJ software.

Assessment of total β-cell mass was performed on scan-stained microscope slides with Inform software using a guinea pig anti-human insulin antibody (DAKO) as the ratio between β-cell surface (µm^2^)/pancreas surface (µm^2^) multiplied by pancreas weight (mg) (24). Assessment of total α-cell mass was performed on scan-stained microscope slides with Inform software using a rabbit anti-glucagon antibody (DAKO) as the ratio between α-cell surface (µm^2^)/pancreas surface (µm^2^) multiplied by pancreas weight (mg). Quantification of insulin-positive or glucagon-positive areas was performed from the entire pancreas on serially cut 8-µm-thick sections. Five to 10 pancreatic sections were processed for immunostaining. Cellular mass obtained for each group was compared using the non-parametric Mann–Whitney statistical test.

### Tetramer Assays and Single-Cell RT-PCR

Tetramers (TMrs) associating the chimeric HLA-A*02:01-H-2K^b^ and the human β2-microglobulin to hPPI peptides were obtained from the NIH Tetramer Core Facility. Stainings were performed as previously reported (11). Incubation of cells with TMrs was performed for 30 min at RT. The following antibodies were used: anti-mouse CD3ε-AlexaFluo700, anti-mouse CD8α-APC, anti-mouse CD19-PerCP-Cy5, and anti-mouse CD14-PercP-Cy5 antibodies (BD/Pharmingen and eBioscience). Cells were analyzed with a BD LSR-Fortessa flow cytometer and the FlowJo 9.2 software (Tree Star Inc.). For single-cell RT-PCR assays, CD8^+^/TMr^+^ T cells were sorted with a FACSAria II at 1 cell/well into 96-well PCR plates containing 5 μl PBS 1× treated with diethyl pyrocarbonate (DEPC) (Sigma) and immediately frozen at −80°C. Single-cell RT-PCR was performed as previously described (11). Perforin (*prf1*), granzyme A (*gzma*), granzyme B (*gzmb*), Fas ligand (*tnfsf6*), IFNγ (*ifng*), TGFβ (*tgfb*), TGFβ R1 (*tgfbr1*), TGFβ R2 (*tgfbr2*), TGFβ R3 (*tgfbr3*), TNFα (*lta*), IL2 (*il2*), IL7R (*il7r*), IL10Rα (*il10ra*), IL15 (*il15*), IL15R (*il15r*), IL21 (*il21*), CCR7 (*ccr7*), and CD3ϵ (*cd3e*) mRNAs were analyzed (**Table S15**). Naive and memory CD8^+^/TMr^+^ T cells were differentiated by the *Foxo1* expression using 5′ (GGA CAG CCG CGC AAG ACC AG) and 3′ (ACT GTT GTT GTC CAT GGA CGC) first PCR primers and 5′ (ATC ACC AAG GCC ATC GAG AGC) and 3′ (TTG AAT TCT TCC AGC CCG CCG A) second PCR primers.

### Islet Isolation Procedure

Islets of Langerhans were isolated as described previously (25). Islets were dissociated with Cell Dissociation Solution Non-Enzymatic (Sigma) for 30 min at RT. Cells were filtered and stained with anti-HLA-DQ-FITC (BD Biosciences), anti-human β2m-PE (BD Biosciences), guinea pig anti-human insulin (DAKO), anti-guinea pig Ig-Biot and SAV-PECy7, anti-mouse CD4-AlexaFluo700, anti-mouse CD8α-APC, anti-mouse CD19-Percp-Cy5.5, and/or anti-mouse TCRβ-efluo450 antibodies (eBioscience). Acquisition was done with a BD FACSAria flow cytometer and analyzed using FlowJo 10.7.1 software.

### Islet Infiltrate Staining

Handpicked islets were pooled in 24-well plates for 24 h in RPMI supplemented with 10% FCS, penicillin–streptomycin, and 5.10^−5^ M β-mercaptoethanol allowing extrusion of infiltrating lymphocytes (25). Infiltrating lymphocytes were recovered. Cells recovered were used for the analysis of T cells and antigen-presenting cells. T-cell analysis was performed using the Foxp3 intra-staining buffer set according to the recommendation of the manufacturer (eBioscience) with the following combination: anti-mouse CD45-efluo450, anti-mouse CD3ϵ-AlexaFluo700, anti-mouse B220-HorizonViolet500, anti-mouse CD8α-Percp-Cy5.5, anti-mouse CD4-PE, anti-mouse Foxp3-APC, and anti-mouse CD25-BrillantViolet711 mAbs (eBioscience). β Cells and dendritic cells were analyzed with anti-mouse CD45-AlexaFluo700, anti-mouse TCRβ-APC-Cy7, anti-mouse B220-HorizonViolet500, anti-mouse CD11b-efluor450, anti-mouse CD11c-APC, anti-mouse CD8α-BrillantViolet605, and anti-mouse CD4-PE mAbs (eBioscience). Acquisitions were performed with a BD LSR-Fortessa flow cytometer and analyzed using FlowJo 10.7.1 software.

### Statistics

The biostatistic method used to compare diabetes incidences between different groups of mice was the log-rank Mantel–Cox test. Comparison of distribution scores between the different mouse strains used the non-parametric Mann–Whitney test. T-cell reactivity was compared using non-parametric Mann–Whitney test and non-parametric Kruskal–Wallis test. ns, non-significant, *p ≤ 0.05, **p ≤ 0.01, ***p ≤ 0.001. For T-cell proliferation assays, we used the Bland and Altman test to determine the threshold value for a global reliability statistic for significant proliferation response.

### Study Approval

Experimental studies were performed in accordance with the Institutional Animal Care and Use Guidelines and accredited by the ethics committee under number CEEA34.CB.024.11. Clinical trial: MESR number DC-2015-2536/IDRCB number 2015-A01875-44.

### Accession Numbers

We submitted the data generated by the Affymetrix SNP Array detection to a public repository (http://www.ncbi.nlm.nih.gov/geo) and the GEO Series accession numbers were GSE101551 and GSE151644.

## Results

### Characterization of the YES-RIP-hB7.1 Mice

In order to enforce the development of T1D in YES mice, we introduced the *hB7.1* gene under the control of the rat insulin gene promoter (RIP) using the LV-RIP-hB7.1 vector (17). We selected three mice that were positive for *hB7.1* for backcrossing onto the YES background. Throughout the crosses, one founder progeny was selected. To further stabilize its lineage, we realized a NimbleGen Sequence Capture of the RIP-hB7.1 transgene of the founder and one of its offspring. Inserted *hB7.1* sequences were identified as indicated in **Table S2**, matching with regions located on chr. 11, 14, 16, and 19 in the founder mouse. Nevertheless, the only region located in chr.19 remained detectable in the offspring, a chromosome with no *Idd* known to associate with TID. We did not evidence hot spots of enhancer transcripts close to the LV-RIP-hB7.1 insertions using the mouse ENCODE database from UCSC Genome Bioinformatics (GSE101551 and GSE151644 GEO data accession numbers). We screened 128 mice from the progeny for the selected insertion sites in order to stabilize the lineage at a homozygote status using a classical progeny test.

### YES-RIP-hB7.1 Mice Develop Spontaneous Type 1 Diabetes

**Figure 1:**
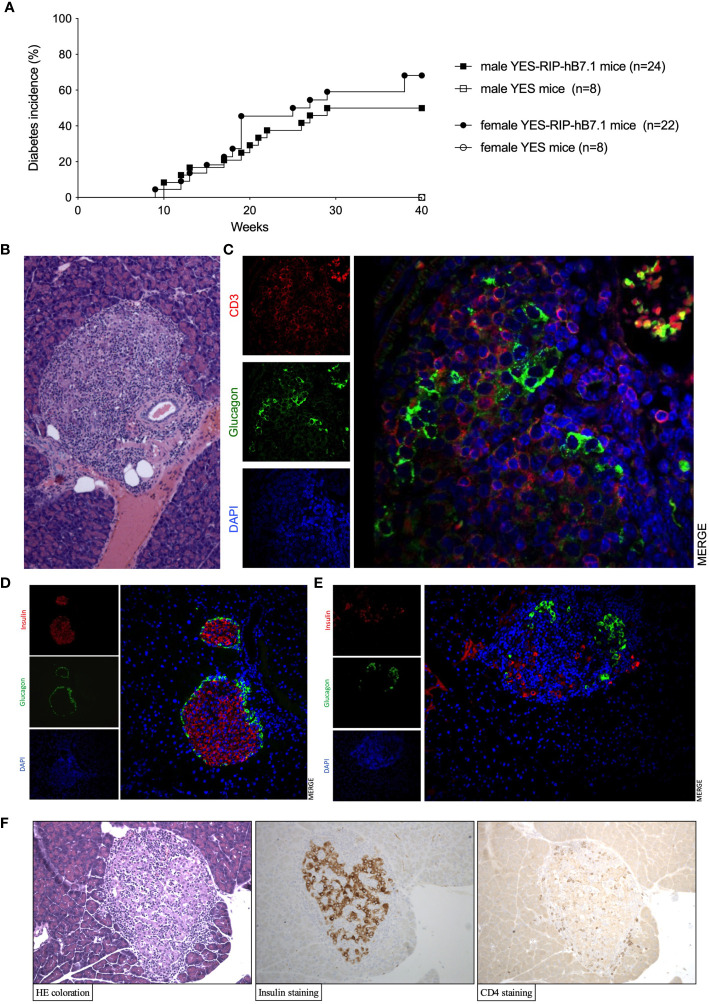
Diabetes incidence, islet infiltration in YES-RIP-hB7.1 mice. **(A)** Diabetes incidence in female (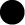, *n* = 22) and male (■, *n* = 24) YES-RIP-hB7.1 mice as compared with female (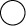, *n* = 8) and male (□, *n* = 6) YES mice as controls. **(B)** Pancreas paraffin sections stained by hematoxylin–eosin in diabetic YES-RIP-hB7.1 mice, ×20. **(C)** Immunofluorescent staining of an islet from a diabetic YES-RIP-hB7.1 mouse with anti-glucagon (green) and anti-CD3ε(red) antibodies, ×40. Immunofluorescent staining of an islet from a diabetic YES mice control **(D)** or YES-RIP-hB7.1 diabetic mouse **(E)** with anti-glucagon (green) and anti-insulin (red) antibodies, ×20. **(F)** Immunohistochemical staining of an islet from a diabetic YES-RIP-hB7.1 mouse with anti-insulin and anti-CD4 antibodies, ×20.

**Figure 2:**
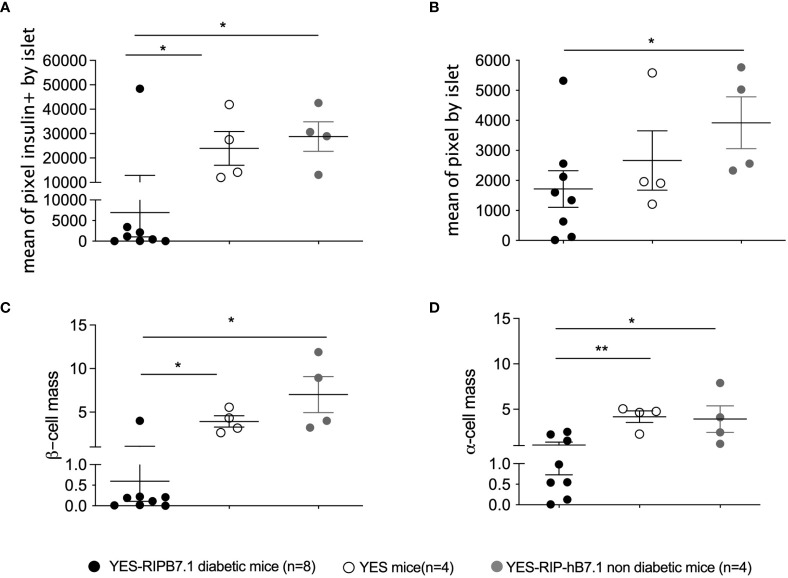
β-Cell and α-cell number and mass in YES-RIP-hB7.1 mice. **(A)** Mean of pixel insulin positive by level of pancreatic section of diabetic YES-RIP-hB7.1 (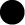, *n* = 8), non-diabetic YES-RIP-hB7.1 (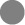, *n* = 4), and control YES (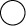, *n* = 4) mice. **(B)** Mean of pixels per islet, representing the estimation of the islet size in pancreas of diabetic YES-RIP-hB7.1 (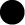, *n* = 8), non-diabetic YES-RIP-hB7.1 (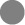, *n* = 4), and control YES (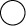, *n* = 4) mice. **(C)** β-Cell mass of diabetic YES-RIP-hB7.1 (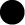, *n* = 8), non-diabetic YES-RIP-hB7.1 (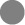, *n* = 4), and control YES (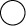, *n* = 4) mice. **(D)** α-Cell mass of diabetic YES-RIP-hB7.1 (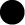), non-diabetic YES-RIP-hB7.1 (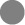), and control YES (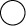) mice. Each dot corresponds to an individual mouse. ns, non-significant, *p ≤ 0.05, **p ≤ 0.01; Mann–Whitney test.

**Figure 3:**
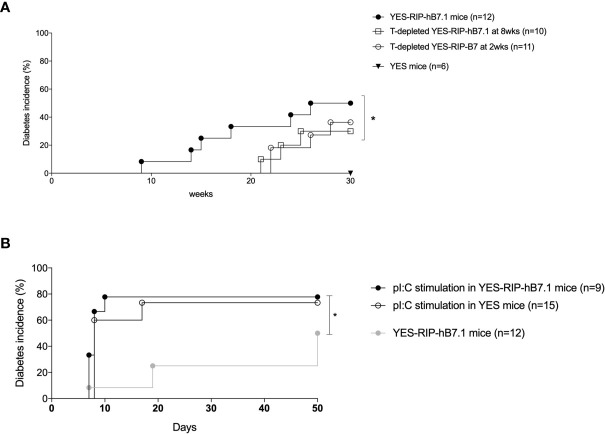
Diabetes incidence after *in vivo* T-cell depletion and poly(I:C) stimulation in YES-RIP-hB7.1 mice. **(A)** Diabetes incidence in untreated YES-RIP-hB7.1 mice (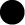, *n* = 12), after *in vivo* T-cell depletion (four i.p. weekly injections of anti-CD4 and anti-CD8 antibodies) in YES-RIP-hB7.1 mice treated from 8 to 11 weeks of age (□, *n* = 10) or from to 2 weeks of age (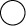, *n* = 11) and in YES mice (▼, *n* = 6) as controls. **(B)** Diabetes incidence in YES-RIP-hB7.1 mice (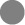, *n* = 12), poly(I:C)-stimulated YES-RIP-hB7.1 mice (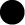, *n* = 15), and poly(I:C)-stimulated YES mice (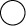, *n* = 15) as controls. PolyI:C stimulation were performed by seven i.p. daily injections of 100 µg pI:C/mice. *p ≤ 0.05; Mann-Withney test.

Insulitis and spontaneous diabetes developed in founder YES-RIP-hB7.1 offspring that were submitted to 20 brother–sister mattings. In the founder progeny, 46 out of 128 YES-RIP-hB7.1 mice developed diabetes, while diabetes was not observed in non-transgenic YES mice. Diabetes incidence of stabilized YES-RIP-hB7.1 mice is shown in **Figure 1A**. Age at onset varied from one mouse to another, spanning from 9 to 51 weeks of age. The overall prevalence of diabetes was similar in female (53.8%) and male (50.0%) YES-RIP-hB7.1 mice. The difference in diabetic incidence was not statistically different between males and females. The average glycemia in YES-RIP-hB7.1 mice when diagnosed as diabetic was 520 ± 115 and 532 ± 99 mg/dl in female and male, respectively. The average glycemia in non-diabetic YES-RIP-hB7.1 mice at the end of the experiment (40 weeks) was 143 ± 35 and 125 ± 18 mg/dl in female and male, respectively. The average glycemia in YES mice was 107 ± 13 and 102 ± 12 mg/dl in female and male, respectively. In order to address whether diabetes was immune-related, we analyzed hematoxylin–eosin-stained pancreas paraffin sections from diabetic and non-diabetic YES-RIP-hB7.1 mice. As shown in **Figure 1B**, insulitis was detected by immunofluorescence staining using an anti-CD3ϵ antibody and a rabbit anti-glucagon antibody to locate remnant islets, showing the infiltration of islets by CD3^+^ T lymphocytes (**Figure 1C**). Glucagon-positive cells were dispersed in remnant islets that showed a dislocated architecture in YES-RIP-hB7.1 diabetic mice (**Figure 1D**) compared with the YES mice control (**Figure 1E**). Stable immunohistochemistry staining with an anti-CD4 antibody confirmed the detection of CD4^+^ T cells within the infiltrate (**Figure 1F**). As shown in **Figure 2**, the number of islets expressing insulin was decreased by 70.9% in diabetic YES-RIP-hB7.1 mice as compared with YES mice or non-diabetic YES-RIP-hB7.1 mice. Islet size (**Figure 2B**) was decreased by over 60% in diabetic YES-RIP-hB7.1 mice as compared with YES controls. A dramatic decrease of the β-cell mass was observed in diabetics YES-RIP-hB7.1 mice as compared with age-matched YES mice (0.60 ± 0.49 *versus* 3.93 ± 0.65, respectively, *p* ≤ 0.02) and non-diabetic YES-RIP-hB7.1 mice (7.01 ± 2.06) (**Figure 2C**). The α-cell mass (**Figure 2D**) also showed a significant decrease in diabetic YES-RIP-hB7.1 mice as compared with non-diabetic YES-RIP-hB7.1 mice and YES controls (0.60 ± 0.49 *versus* 3.93 ± 0.65, respectively, *p* ≤ 0.02). In non-diabetic YES-RIP-hB7.1 mice, the α-cell mass was heterogeneous (7.01 ± 2.06). Infiltrates from three diabetic mice were recovered and pooled to be analyzed (**Figure S1**). They were composed of 52% T cells, among which 81% were CD8^+^ T cells, 8% were CD4^+^ T cells, 21% were β cells, 0.8% were dendritic cells, and 0.6% were macrophages. Among CD4^+^ T cells, 7% were CD4^+^CD25^+^FoxP3^+^ T cells. Infiltrates recovered from three non-diabetic YES-RIP-hB7.1 mice (**Figure S1**) were composed of 56% T cells, among which 56% were CD8^+^ T cells, 34% were CD4^+^ T cells, 34% were β cells, and 2.3% were dendritic cells or macrophages. Among CD4^+^ T cells, 1% were CD25^+^FoxP3^+^. These data demonstrate the presence of an immune response along the development of diabetes in YES-RIP-hB7.1 mice, a dramatic decrease in β cells and a reduced α-cell mass. As the extent of infiltration was milder than the infiltrate seen in the NOD mouse model, we addressed whether autoimmune development could be delayed by a transient treatment with anti-CD4 and anti-CD8 monoclonal antibodies. As shown in **Figure 3A**, *in vivo* depletion of T cells by anti-CD4 and anti-CD8 antibodies from either 2 to 5 or 8 to 11 weeks of age significantly delayed T1D development in YES-RIP-hB7.1 mice. There was no difference in the protection observed in early-treated and late-treated mice. As previously described in the YES mouse model (16), we addressed whether diabetes development could be triggered in YES-RIP-hB7.1 mice by poly(I:C). As shown in **Figure 3B**, diabetes was induced within 6 to 17 days following the first poly(I:C) injection.

### hPPI-Specific CD8^+^ T lymphocytes Are Detected in YES-RIP-hB7.1 Mice

**Figure 4:**
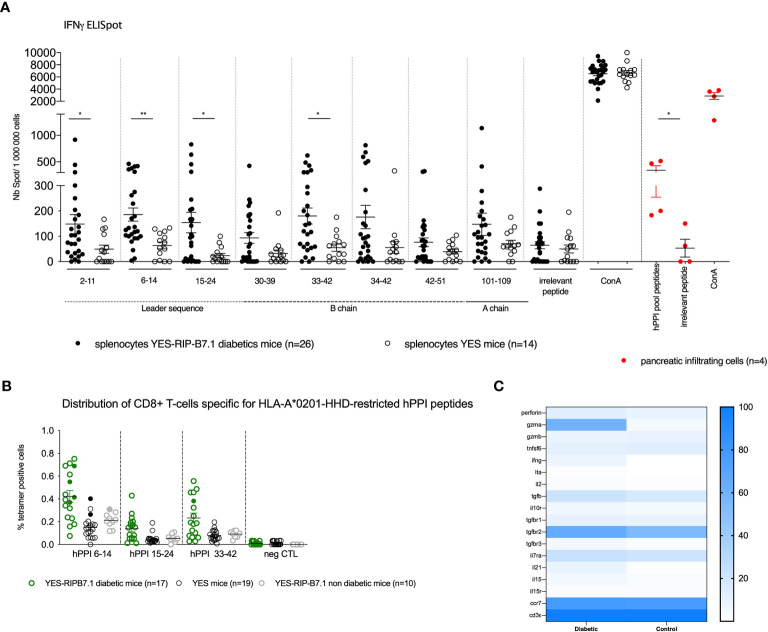
hPPI responses of YES-RIP-hB7.1 CD8^+^ T cells. **(A)** IFNγ responses to hPPI peptides. Splenocytes from individual diabetic (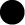), non-diabetic (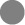) YES-RIP-hB7.1 mice, and control YES mice (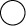) were restimulated overnight with HLA-A*02:01-restricted hPPI peptides (20 µg/ml). Red cells correspond to cells from pancreatic infiltration. Each dot corresponds to an individual mouse for each peptide studied. **(B)** hPPI-specific CD8^+^ T-cell expansions in YES-RIP-hB7.1 mice. Splenic CD8^+^ T cells were stained with hPPI_6-14_, hPPI_15-24_, and hPPI_33-42_ or control tetramers comparing diabetic (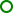), non-diabetic YES-RIP-hB7.1 mice (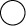), and control YES mice (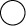). Each dot corresponds to an individual mouse. Filled symbols correspond to the sorting cells for multiparametric RT-PCR. **(C)** Heat map of single-cell multiparametric RT-PCR in hPPI_6-14_, hPPI_15-24_, or hPPI_33-42_-specific CD8^+^ T cells from diabetic YES-RIP-hB7.1 or non-diabetic YES-RIP-hB7.1 mice and control YES mice. ns, non-significant, *p ≤ 0.05, **p ≤ 0.01; Mann–Whitney test.

We previously characterized preproinsulin-specific CD8^+^ T cells in human T1D diabetes (11). In order to validate the YES-RIP-hB7.1 mice as a model to study T cells in T1D, we characterized CD8^+^ T-cell responses in 12- to 20-week-old diabetic mice and control mice that remained diabetes-free up to 30 weeks of age as previously reported (11). Using an IFNγ-ELISpot assay, we evaluated CD8^+^ T-cell responses to a library of hPPI peptides that were selected for presentation by HLA-A*02:01. Most hPPI peptides that were previously defined as recognized by human CD8^+^ T cells (11, 26) were recognized by T cells obtained from diabetic YES-RIP-hB7.1 mice (**Figure 4A**). Responses were detected against hPPI_2-11_ (*p* ≤ 0.03), hPPI_6-14_ (*p* ≤ 0.001), hPPI_15-24_ (*p* ≤ 0.02), and hPPI_33-42_ (*p* ≤ 0.013). No responses were observed against any of these hPPI peptides in control YES mice and in non-diabetic YES-RIP-hB7.1 mice. When considering responses that were over the mean ± 3SD of responses seen in control YES mice, significant responses were observed against hPPI_6-14_ (9/26, *p* ≤ 0.016), hPPI_15-24_ (11/26, *p* ≤ 0.004), and hPPI_33-42_ (9/26, *p* ≤ 0.016). Considering individual mice, responses were observed against hPPI_2-11_ (19.2%), hPPI_30-39_ (23.1%), hPPI_34-42_ (19.2%), hPPI_42-51_ (15.4%), and hPPI_101-109_ (15.4%), although they did not reach statistical significance in the whole population of diabetic mice analyzed (**Table S3**). As a whole, 93.75% YES-RIP-hB7.1 diabetic mice showed a response to at least one of the HLA-A*02:01-restricted hPPI peptides studied (defined as > mean ± 3SD of the response of YES mice against each given peptide, **Table S3**). An IFNγ-ELISpot response of pancreatic infiltrating cells was also documented against pooled hPPI peptides (**Figure 4A**, in red symbol) as the limited number of infiltrating cells prevented testing individual peptides. Using chimeric HLA-A*02:01 tetramers, we detected significant expansions of CD8^+^ T cells against hPPI_6-14_ (47.1%), hPPI_15-24_ (29.4%), and hPPI_33-42 (_47.1%) in YES-RIP-hB7.1 mice as compared with YES mice (**Figure 4B**). Considering individual mice, an expansion of CD8^+^ T cells was seen in 70.6% YES-RIP-hB7.1 mice against either hPPI_6-14_, hPPI_15-24_, or hPPI_33-42_. Single hPPI-specific CD8^+^ T cells showed gene expression profiles that were characteristic of memory T cells (**Figure 4C**). hPPI-specific CD8^+^ T cells from diabetic YES-RIP-hB7.1 mice expressed *gzmA* (59.38%), among which 100% co-expressed *ccr7*, while only 4.33% hPPI-specific CD8^+^ T cells from both YES-RIP-hB7.1 non-diabetic mice and YES control mice expressed *gzmA*, of which 76.7% co-expressed *ccr7*. To confirm a naive T-cell profile of cells from non-diabetic mice, we performed gene expression analysis for *foxo1*, which is expressed in basal naive T cells (27). An increased percentage of CD8^+^ T cells expressed *foxo1* in non-diabetic as compared with diabetic mice (**Figure S2A**). A significant level of *foxo1* expression was detected in 10-cell and single-cell batches (**Figure S2B**) in non-diabetics YES-RIP-hB7.1 mice when normalized to *cd3ϵ* expression. Finally, we determined *ex vivo* cytotoxic effects of hPPI-specific CD8^+^ T cells (**Figure S3**) in five diabetic YES-RIP-hB7.1 mice as compared with three YES controls in a LDH-release assay using peptide-pulsed HLA-A*02.01-transfected P815 target cells. Cytotoxic responses were detected against all hPPI peptides in diabetic YES-RIP-hB7.1 mice. Mice considered individually responded to different hPPI peptides. All mice that responded against at least one hPPI peptide also responded to full-length hPPI. Three mice showed cytotoxic responses against several hPPI peptides and two mice showed no cytotoxicity against any of the eight hPPI peptides. A heterogeneity of the cytotoxic responses in diabetic YES-RIP-hB7.1 was observed against hPPI.

### hPPI-Specific CD4^+^ T-Cell Responses in YES-RIP-hB7.1 Mice

**Figure 5:**
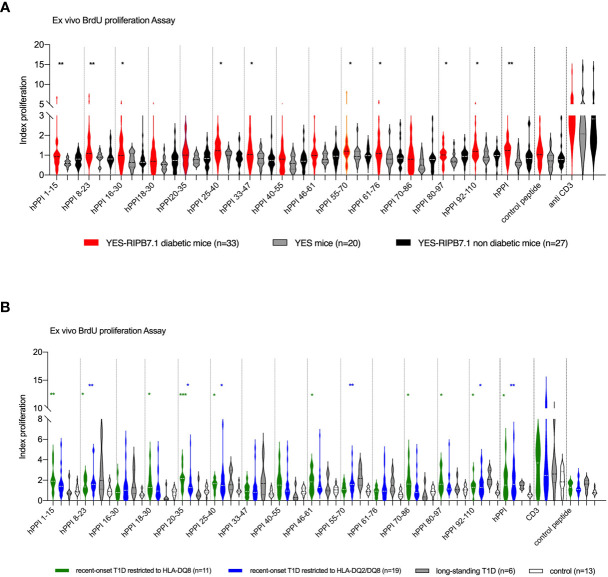
CD4^+^ T-cell responses against hPPI in YES-RIP-hB7 mice and T1D patients. **(A)** Proliferative responses to full-length hPPI and hPPI peptides hPPI_1-15_, hPPI_8-23_, hPPI_16-30_, hPPI_18-30_, hPPI_20-35_, hPPI_25-40,_ hPPI_33-47_, hPPI_40-55_, hPPI_46-61_, hPPI_55-70_, hPPI_61-76_, hPPI_70-86_, hPPI_80-97_, and hPPI_92-110_. Splenocytes from diabetic YES-RIP-hB7.1 (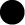), non-diabetic YES-RIP-hB7.1 (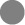), and YES mice (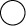) were stimulated *in vitro* for 3 days, and proliferation was evaluated by measuring BrdU incorporation. **(B)** Proliferative responses to full-length hPPI and hPPI peptides hPPI_1-15_, hPPI_8-23_, hPPI_16-30_, hPPI_18-30_, hPPI_20-35_, hPPI_25-40_, hPPI_33-47_, hPPI_40-55_, hPPI_46-61_, hPPI_55-70_, hPPI_61-76_, hPPI_70-86_, hPPI_80-97_, and hPPI_92-110_. PBMCs from new-onset T1D patients restricted to HLA-DQ8 (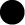) or restricted to HLA-DQ2/DQ8 (■), long-standing T1D patients (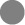), and controls (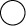) were stimulated *in vitro* for 3 days, and proliferation was evaluated by measuring BrdU incorporation. Statistic significances were obtained with data from control compared with HLA-DQ8 restriction patients (black bar) or with HLA-DQ2/DQ8 restriction patients (gray bar). Each dot represents individual mouse. *p ≤ 0.05, **p ≤ 0.01, ***p ≤ 0.001; Mann–Whitney test.

We studied HLA-DQ8-restricted T-cell responses to an overlapping hPPI peptide library. Significant T-cell proliferative responses were detected against hPPI_1-15_ (*p* ≤ 0.003), hPPI_8-23_ (*p* ≤ 0.008), hPPI_16-30_ (*p* ≤ 0.015), hPPI_25-40_ (*p* ≤ 0.014), hPPI_33-47_ (*p* ≤ 0.05), hPPI_55-70_ (*p* ≤ 0.029), hPPI_61-76_ (*p* ≤ 0.032), hPPI_80-97_ (*p* ≤ 0.022), and hPPI_92-110_ (*p* ≤ 0.028) peptides in YES-RIP-hB7.1 diabetic mice as compared with YES controls (**Figure 5A**). A non-significant trend for proliferative responses was detected against the same peptides in a few non-diabetic YES-RIP-hB7.1 mice. As in the case of CD8^+^ T cells, individual responses were observed against peptides covering the whole hPPI sequence. After determination of the threshold value for each peptide in controls using pairs of measurements and the Bland and Altman test (**Figure S4**, **Table S4**), proliferative responses were observed in a significant number of mice against all peptides except for hPPI_16-30_, hPPI_40-55_, and hPPI_46-61_. A significant response to full-length hPPI was observed in 51.5% of mice (*p* ≤ 0.009). Significant responses were observed in more than 30% diabetic YES-RIP-hB7.1 mice against hPPI_1-15_ (30.3% mice, *p* ≤ 0.008), hPPI_8-23_ (36.4% mice, *p* ≤ 0.002), hPPI_18-30_ (30.3% mice, *p* ≤ 0.04), hPPI_25-40_ (36.4% mice, *p* ≤ 0.002), hPPI_33-47_ (30.3% mice, *p* ≤ 0.009), hPPI_55-70_ (39.4% mice, *p* ≤ 0.009), hPPI_61-76_ (30.3% mice, *p* ≤ 0.04), hPPI_70-86_ (30.3% mice, *p* ≤ 0.04), and hPPI_92-110_ (45.4% mice, *p* ≤ 0.002) (**Table S5**). We observed a significant correlation between proliferative responses against the hPPI protein and several hPPI peptides in diabetic YES-RIP-hB7.1 mice, including hPPI_16-30_, hPPI_25-40_, hPPI_33-47_, hPPI_55-70_, hPPI_61-76_, and hPPI_92-110_ (**Figure S5**). Proliferative responses were seen against at least one hPPI peptide in 87.9% diabetic mice.

### hPPI-Specific CD4^+^ T Lymphocytes Are Detected in the Peripheral Blood of T1D Patients

The presence of hPPI-specific CD4^+^ T cells in the peripheral blood of T1D diabetic patients was analyzed by *ex vivo* proliferative assay and compared with HLA-DQ8 healthy donor and type 2 diabetes (T2D) patients as controls in order to address whether hPPI regions recognized in human matched with peptides recognized in YES-RIP-hB7.1 mice. Clinical features of T1D patients and control subjects (who were negative for detection of anti-GAD, anti-IA2, and anti-ZnT8 autoantibodies) are summarized in **Table 1**. Considering the possible trans-complementation between HLA-DQ8 and HLA-DQ2 class II MHC molecules, T1D patients were included according to their HLA-DQ8 or HLA-DQ2/DQ8 genotype. A proliferative response was observed against full-length recombinant hPPI in HLA-DQ8 T1D patients (*p* ≤ 0.04) and in HLA-DQ2/DQ8 T1D patients (*p* ≤ 0.003) as compared with controls (**Figure 5B**). When dissecting epitopes recognized, significant proliferative responses were observed against leader sequence hPPI_1-15_ (*p* ≤ 0.007), hPPI_8-23_ (*p* ≤ 0.003) and hPPI_18-30_ (*p* ≤ 0.02) peptides, overlapping leader sequence and B-chain hPPI_20-35_ peptide (*p* ≤ 0.007), B-chain hPPI_25-40_ (*p* ≤ 0.03) and hPPI_46-61_ (*p* ≤ 0.03) peptides, C-peptide hPPI_70-86_ (*p* ≤ 0.05) peptide, overlapping C-peptide and A-chain hPPI_80-97_ peptide (*p* ≤ 0.02), and the A-chain hPPI_92-110_ peptide (*p* ≤ 0.03) in HLA-DQ8 patients (**Figure 4B**). Significant proliferative responses were observed against leader sequence hPPI_8-23_ peptide (*p* ≤ 0.009), B-chain hPPI_20-35_ (*p* ≤ 0.05) and hPPI_25-40_ (*p* ≤ 0.035) peptides, C-peptide hPPI_55-70_ (*p* ≤ 0.003) peptide, and A-chain hPPI_92-110_ (*p* ≤ 0.04) peptide in HLA-DQ2/DQ8 T1D patients. After determination of the threshold value for each peptide in controls using pairs of measurements and the Bland and Altman test (**Figure S6**, **Table S6**), proliferative responses against hPPI_1-15_ (72.75%, *p* ≤ 0.01), hPPI_18-30_ (45.4%, *p* ≤ 0.041), hPPI_20-35_ (72.7% of T1D, *p* ≤ 0.04), hPPI_40-55_ (54.4%, *p* ≤ 0.003), hPPI_46-61_ (54.4%, *p* ≤ 0.02), and hPPI_80-97_ (63.6%, *p* ≤ 0.03) were seen in a significant number of HLA-DQ8 recent-onset T1D patients (**Table S7**). Significant proliferative responses to hPPI_33-47_ (42.1%, *p* ≤ 0.05), hPPI_40-55_ (36.8%, *p* ≤ 0.03), hPPI_55-70_ (57.9%, *p* ≤ 0.0006), and hPPI_92-110_ (47.4%, *p* ≤ 0.03) were observed in HLA-DQ2/DQ8 recent-onset T1D patients (**Table S8**). Distribution of proliferative responses to hPPI peptides was different in HLA-DQ8 and HLA-DQ2/DQ8 patients. As a total, proliferative responses were seen against at least one hPPI peptide in 94.44% T1D patients. Overall, the proliferative responses in HLA-DQ8 T1D patients largely matched the responses in YES-RIP-hB7.1 mice.

### Reactivity Against Spliced hPPI in YES-RIP-hB7.1 Mice and HLA-DQ8 and DQ2/DQ8 Patients

**Figure 6:**
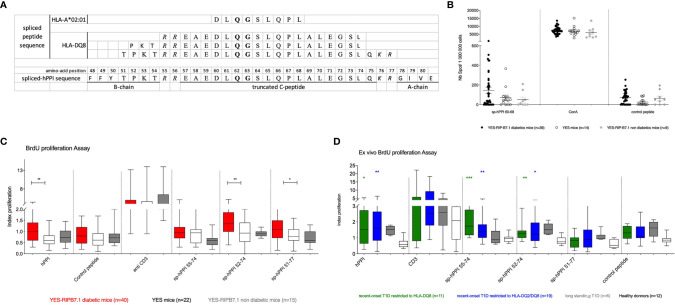
Responses to spliced hPPI. **(A)** Sequence of the spliced hPPI protein and spliced peptides restricted to HLA-A*02:01 and HLA-DQ8. Bold delimitates the truncated region of the alternatively spliced insulin protein sequence and italic defines bond-disulfur sites. **(B)** IFNγ responses to spliced hPPI peptides restricted to HLA-A*02:01. Splenocytes from individual diabetic (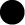), non-diabetic (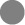) YES-RIP-hB7.1 mice, and control YES mice (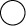) were restimulated overnight with sp-hPPI_60-68_ peptide (20 µg/ml). Each dot corresponds to an individual mouse for each condition studied. **(C)** Proliferative responses to sp-hPPI_55-74_, sp-hPPI_52-74_, and sp-hPPI_51-77_ peptides. Splenocytes from diabetic YES-RIP-hB7.1 (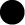), non-diabetic YES-RIP-hB7.1 (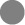), and YES mice (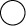) were stimulated *in vitro* for 3 days, and proliferation was evaluated by measuring BrdU incorporation. **(D)** Proliferative responses to sp-hPPI_55-74_, sp-hPPI_52-74_, and sp-hPPI_51-77_ peptides. PBMC from new-onset T1D patients restricted to HLA-DQ8 (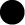) or restricted to HLA-DQ2/DQ8 (■), long-standing T1D patients (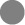), and controls (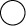) were stimulated *in vitro* for 3 days, and proliferation was evaluated by measuring BrdU incorporation. Statistic significances were obtained with data from control compared with HLA-DQ8 restriction patients (black bar) or with HLA-DQ2/DQ8 restriction patients (gray bar). Each dot represents individual mouse. *p ≤ 0.05, **p ≤ 0.01, ***p ≤ 0.001; Mann–Whitney test.

Based on the observation of insulin mRNA alternative splicing events when reanalyzing databases generated in the context of human islet inflammation (28, 29), we designed an HLA-A*02:01-restricted spliced hPPI peptide, sp-hPPI_60-68_, and three HLA-DQ8-restricted spliced peptides, named sp-hPPI_55-74_, sp-hPPI_52-74_, and sp-hPPI_51-77_ using HLA-restrictor and NetMHCII 2.3 server, respectively (**Figure 6A**). Using an IFNγ-ELISpot assay, we detected HLA-A*02:01-restricted IFNγ responses against sp-hPPI_60-68_ peptide (**Figure 6B**) and HLA-DQ8-restricted responses to the sp-hPPI_51-77_ peptide (*p* ≤ 0.05) in splenocytes from diabetic YES-RIP-hB7.1 mice as compared with non-diabetic YES-RIP-hB7.1 or YES mouse controls (**Figure 6C**). Proliferative responses were further observed against the sp-hPPI_52-74_ and sp-hPPI_51-77_ peptides (*p* ≤ 0.009 and *p* ≤ 0.049, respectively, **Figure 6C**) in diabetic YES-RIP-hB7.1 mice as compared with non-diabetic YES-RIP-hB7.1 or YES controls. Seemingly, we addressed whether proliferative responses were detected in T1D patient PBMCs. Significant proliferative responses were seen against the sp-hPPI_55-74_ and sp-hPPI_52-74_ peptides in recent-onset HLA-DQ8 patients (*p* ≤ 0.0001 and *p* ≤ 0.001, respectively, black circle, **Figure 6D**) and in recent-onset HLA-DQ2/DQ8 diabetic patients (*p* ≤ 0.008 and *p* ≤ 0.03, respectively, black square, **Figure 6D**) as compared with control donors. Threshold values were determined in YES mice (**Table S9**) and in human controls (**Table S10**) using pairs of measurements and the Bland and Altman test (**Figure S7**). The frequency of recognition of sp-hPPI_55-74_, sp-hPPI_52-74_, and sp-hPPI_51-77_ peptides was not significantly different in diabetic YES-RIP-hB7.1 mice (**Table S11**). By contrast, the frequency of recognition of sp-hPPI_55-74_ and sp-hPPI_52-74_ peptides was significant in recent-onset HLA-DQ8 (57.9%, *p* ≤ 0.009 and 52.6%, *p* ≤ 0.02, respectively, **Table 2**) and HLA-DQ2/DQ8 (63.6%, *p* ≤ 0.009 and 72.5%, *p* ≤ 0.003, respectively, **Table 2**) T1D patients.

**Table 2:** Anti-spliced hPPI proliferative responses in T1D HLA-DQ8 patients.

	T1D patients restricted to HLA-DQ8						
Peptide	Responses (proliferation index)				Frequencies of recognition (mean ± 3SD)		
	Recent-onset T1D patients (*n* = 11)	Controls (*n* = 12)	*p*-value		T1D patients (*n* = 11)	Controls (*n* = 12)	*p*-value
sp-hPPI_55-74_	1.92 (1–3.48)	0.95 (0.65–1.49)	≤0.0001		7/11	1/12	≤0.009
sp-hPPI_52-74_	1.35 (0.77–2.86)	0.82 (0.51–1.32)	≤0.002		8/11	1/12	≤0.003
sp-hPPI_51-77_	0.82 (0.2–1.6)	0.61 (0.33–1.01)			2/11	0/12	
	T1D patients restricted to HLA-DQ2/DQ8						
Peptide	Responses (proliferation index)			Frequencies of recognition (mean ± 3SD)			
	Recent-onset T1D patients (*n* = 19)	Controls (*n* = 12)	*p*-value	T1D patients (*n* = 19)		Controls (*n* = 12)	*p*-value
sp-hPPI_55-74_	1.58 (0.6–4.64)	0.82 (0.51–1.32)	≤0.008	11/19		1/12	≤0.02
sp-hPPI_52-74_	1.44 (0.4–4.03)	0.82 (0.51–1.32)	≤0.03	10/19		1/12	≤0.02
sp-hPPI_51-77_	1.08 (0.12–3.21)	0.61 (0.33–1.01)		6/19		0/12	

## Discussion

We developed a new preclinical model of spontaneous T1D in YES mice engineered to express the human co-activation hB7.1 gene in β cells by injecting a HIV-derived recombinant lentiviral vector in which a RIP-hB7.1 transgene has been inserted as previously reported (17), in addition to expression of human MHC and insulin genes instead of the corresponding mouse genes (16). We obtained a founder in which four insertions were detected and one stabilized as homozygous in the progeny. This stable insertion was located at a distance from any known Idd loci. The YES genetic background on which the C57BL/6 background is dominant was previously described (16). This likely indicates that the main genetic constraint that favors diabetes development, beyond expression of hB7.1, is the expression of the human class II HLA-DQ8 and, to a lower extent, class I HLA-A*02:01 alleles. While less than 2% conventional RIP-hB7.1 transgenic mice developed diabetes by 8 months of age (30), spontaneous diabetes was commonly observed in transgenic mice that co-expressed RIP-hB7.1 and the human insulin gene β cells (12, 31). Co-expression of RIP-hB7.1 in addition to HLA-DQ8 has been shown to allow the development of diabetes on a C57BL/6 genetic background (32–35). Along with the development of diabetes and islet infiltration by CD4^+^ and CD8^+^ T cells, a dramatic decrease of β-cell mass and a decrease in α-cell mass were observed in diabetic YES-RIP-hB7.1 mice, as previously been reported in NOD mice (36). In addition, RNA sequencing of human islet cells obtained from T1D patients showed a decrease in the expression of glucagon and other α-cell genes (37, 38), which is confirmed by a recent study on T1D patients based on the network for Pancreatic Organ Donors repository (39). In diabetic YES-RIP-hB7.1 mice in which the islet infiltrate was recovered from the pancreas, the islet infiltrate was predominantly composed of lymphocytes as observed in human T1D insulitis (40). CD8^+^ T cells were largely predominant, suggesting that they were a driving force in the diabetes process. We previously reported an increased percentage of single positive CD8^+^ T cells in the YES mouse, likely related to a lower efficiency of class II HLA-DQ8 to select CD4^+^ T cells than of class I HLA-A*02:01 to select CD8^+^ T cells (10). However, CD4^+^ T cells were recovered from non-diabetic YES-RIP-hB7.1 infiltrates in addition to CD8^+^ T cells. Spontaneous diabetes was significantly delayed in YES-RIP-hB7.1 mice following transient treatment with depleting anti-CD4 and anti-CD8 monoclonal antibodies, leaving open the issue of the predominant role of either CD4^+^ or CD8^+^ T cells in our model. In most T1D preclinical models, CD4^+^ T cells have been reported as dominant although a major role of CD8^+^ T cells has been reported in some models (41). In addition, an acute form of diabetes was induced in 8-week-old, prediabetic, YES-RIP-hB7.1 mice by seven daily poly(I:C) injections, as reported in the YES funders (16), indicating that different triggering events may concur to autoimmune T1D, as is probably the case in human disease. These data are reminiscent of data involving T1D induction by Coxsackie B4 virus (42–44), pointing to islet–environment interactions through signals carried by pattern recognition receptors (PPRs) (45, 46) in induction of T1D (47, 48).

In YES-RIP-hB7.1 mice, CD8^+^ T cells were detected against a wide array of epitopes covering the hPPI sequence. Responses were observed against two leader sequence epitopes, i.e., hPPI_6-14_ and hPPI_15-24_, and the B-chain peptide hPPI_33-42_ using an IFNγ-ELISpot assay, as was previously reported in human (11, 49). We and others have previously provided evidence that they are naturally processed as hPPI_6-14_-, hPPI_15-24_-, and hPPI_33-42_-specific CD8^+^ T cells are detected (11, 50), which are cytotoxic either to hPPI-HLA-A*02:01 doubled transfected-P815 target cells (26) or to human HLA-A*02:01 islets (49). As in human, however, the repertoire of hPPI epitopes recognized by CD8^+^ T cells was highly diversified from one mouse to another, with CD8^+^ T-cell responses observed against a wide array of hPPI epitopes spanning the whole hPPI sequence, beyond the three aforementioned epitopes. No correlation was observed between the affinity of peptides for soluble HLA-A*02:01 (11, 21, 26) and the prevalence of CD8^+^ T-cell responses in diabetic mice. Using HLA-A*02:01-restricted tetramers, we further detected expansions of CD8^+^ T cells against the three dominant HLA-A*02:01-restricted hPPI peptides. An expansion of hPPI-specific hPPI_6-14_-, hPPI_15-24_-, and hPPI_34-42_-CD8^+^ T cells was seen in diabetic mice and in some non-diabetic mice, as we also reported in human T1D. The study of gene expression in hPPI_6-14_-, hPPI_15-24_-, and hPPI_33-42_-specific CD8^+^ T cells allowed discriminating diabetic mice, in which 40% to 80% hPPI-specific single CD8^+^ T cells expressed *gzma*, from non-diabetic YES-RIP-hB7.1 mice, in which *gzma* expression was absent. Fifty-five percent to 95% CD8^+^ T cells were shown to express *ccr7*, suggesting that CD8^+^ T cells were mostly central memory cells in diabetic YES-RIP-hB7.1 mice, while CD8^+^ T cells mostly showed a naive phenotype in non-diabetic YES-RIP-hB7.1 mice, as confirmed by increased expression of *foxo1*, a gene that has been implicated in the regulation of T-cell homeostasis (51).

The study of class I HLA-A*02:01-restricted CD8^+^ T-cell responses in YES-RIP-hB7.1 diabetic mice has shown to broadly match epitope recognition that we previously reported in HLA-A*02:01 T1D patients. The YES-RIP-hB7.1 model is thus likely relevant to study recognition of a major human β-cell autoantigen, namely preproinsulin, presented by human susceptibility MHC molecules. In contrast with class-I-restricted epitopes, HLA-class II-restricted CD4^+^ T-cell responses remain ill-defined (52). CD4^+^ T-cell responses were observed in diabetic YES-RIP-hB7.1 mice against full-length hPPI and a wide array of hPPI peptides spanning the whole hPPI sequence, both in mouse and human, without any evidence for recognition of a dominant region. This is in contrast with previous reports of selective IAg7-restricted and HLA-DQ8-restricted T-cell responses observed in NOD mouse (7, 53, 54) and in human (7) against the B-chain peptide B9-23 (hPPI_33-47_). In NOD mouse, although an insulin B-chain epitope has been proposed as dominant along the autoimmune response to β cells, we showed that hybridomas directly generated from the islet-infiltrating CD4^+^ T cells recognized a large array of insulin epitopes (25). Significant frequencies of proliferative responses were observed against hPPI_1-15_, hPPI_33-47_, hPPI_70-86_, and hPPI_92-110_ peptides in both T1D induced by polyI:C in YES mice and spontaneous T1D observed in YES-RIP-hB7.1 mice. YES-RIP-hB7.1 mice showed significant proliferative responses to additional hPPI peptides, possibly reflecting an immune heterogeneity that is likely to apply to human disease (55, 56). Proliferative responses were observed against hPPI_1-15_, hPPI_8-23_, hPPI_25-40_, hPPI_80-97_, and hPPI_92-110_ both in HLA-DQ8 T1D patients and in diabetic YES-RIP-hB7.1 mice (16). In previous studies, epitopes located in the C-peptide region have been characterized as recognized by T-cell clones obtained from patients with T1D (57, 58). The hPPI_55-69_ epitope located in the junction of B-chain and C-peptide region was identified as a deaminated proinsulin peptide with cross-reactivity with native proinsulin peptide upon restimulation (59). Epitopes located in the peptide signal region and in the B-chain–C-peptide overlapping region have been identified as natural epitopes presented by high-risk HLA-DQ2/DQ8 heterozygous molecules (60, 61). HLA-DQ8-restricted CD4^+^ T-cell clones obtained from a T1D pancreatic infiltrate have been shown to recognize an hPPI epitope located in the C-peptide (57) that covers hPPI_61-76_ and hPPI_80-97_ for which we found responses in YES-RIP-hB7.1 mice and HLA-DQ8 T1D patients. We further found responses to hPPI_61-76_ in diabetic YES mice upon polyI:C injections (16). CD4^+^ T cells specific of the InsB30-C13 peptide (hPPI_55-69_) have been reported in 68% of HLA-DQ2/DQ8-restricted T1D patients *versus* 37% in healthy donors (59). This epitope is similar to our hPPI_55-70_ epitope for which we found significant proliferative responses in spontaneous-diabetic YES-RIP-hB7.1 mice (39.4%) and in T1D HLA-DQ2/DQ8 patients (57.9% in T1D patients *versus* 0% in control). In our study, HLA-DQ8-restricted patients showed responses against signal peptide hPPI epitopes which were not observed in HLA-DQ2/DQ8 patients. Reactivity against the B-chain or A-chain was observed in both HLA-DQ8 and HLA-DQ2/DQ8 patients. C-peptide recognition was mostly observed in HLA-DQ2/DQ8 patients. The frequency of recognition of hPPI peptides observed in diabetic YES-RIP-hB7.1 mice showed similarly significant responses against the hPPI signal peptide.

According to the description of spliced epitopes generated along inflammation development in the islets of Langerhans (62), we addressed whether T cells were responsive to these neoepitopes. Following evidence for CD4^+^ T-cell recognition of hPPI-spliced peptides in diabetic YES-RIP-hB7.1 mice, we evaluated corresponding responses in T1D patients. As in the mouse, we observed responses in both HLA-DQ8 and HLA-DQ2/DQ8 patients against an hPPI-spliced sequence that joins spliced hPPI_55-74_ and spliced hPPI_52-74_ peptides. This points to the interest of the YES-RIP-hB7.1 model in the study of modified hPPI peptides, which will be interesting to explore for other modifications such as citrullinated peptides (63) or epitopes issued from defective ribosomal insulin gene products (64).

Mouse models have been developed that express T1D susceptibility HLA class I (65, 66) or class II genes (31, 67). They allow defining epitopes on murine autoantigens that possibly correspond to epitopes recognized on human autoantigen along human T1D (37, 54). Sequence differences between murine and human MHC presenting molecules cannot exclude, however, that sets of epitopes defined on murine autoantigen differ from those recognized in human T1D. Models have further been reported that express T1D susceptibility HLA class I A*02:01 and/or the high-susceptibility *DQ8* class II gene along with either the human *preproinsulin* or *GAD* genes (68, 69). These models are likely to allow characterizing autoantigen epitopes that may directly apply to human T1D (11, 70). Among these models, the YES-RIP-hB7.1 mouse is expected to allow the characterization of hPPI-specific CD8^+^ and CD4^+^ hPPI epitopes on a major autoantigen targeted in T1D, including new epitopes, such as spliced or modified epitopes, in this proinflammatory context (70, 71). Beyond allowing the identification of HLA-A02*01 and DQ8-restricted epitopes, it allows exemplifying different mechanisms of induction of T1D in the context of human disease that is likely heterogeneous (55, 56). Such models may prove valuable in developing T-cell assays in T1D and evaluating strategies to induce immune tolerance in T1D patients using peptides targeted by the autoimmune response to β cells.

## Data Availability Statement

The original contributions presented in the study are publicly available. These data can be found here: http://www.ncbi.nlm.nih.gov/geo, GSE101551 and GSE151644.

## Ethics Statement

The studies involving human participants were reviewed and approved by the Research Ministry Authorization MESR under number DC-2015-2536/IDRCB number 2015-A01875-44. The patients/participants provided their written informed consent to participate in this study. The animal study was reviewed and approved by the Ethics Committee n°34 of Paris Descartes under number CEEA34.CB.024.11.

## Author Contributions

SL performed the experiments, was involved in the discussion, and contributed to the writing of the manuscript. SG was in charge of the transgenic animals. AG performed DAB staining. FLet and MV were involved in Affymetrix genotyping array discussion and RIP-hB7.1 capture strategy. PN and MB performed big data analysis. PC participated in the production of lentiviral particles for the lentiviral transgenesis. EL was responsible for patient recruitment and follow-up. MC and DE were implicated and collaborated in the alternative spliced hPPI section. FLem was involved in the discussion and manuscript editing. CB designed the experiments, chaired discussions, and wrote the manuscript. All authors contributed to the article and approved the submitted version.

## Funding

This work was performed within the Département Hospitalo-Universitaire (DHU) AUToimmune and HORmonal diseaseS and supported by ANR grant R11189KK-RPV11189KKA, ANR2010-Biot-00801, EFSD grant 1-2008-106, and INNODIA grant E15179KK. DE was supported by grants from the Fonds National de la Recherche 420 Scientifiques (FNRS) - Welbio CR-2015A-06, Belgium, and the Innovative Medicines Initiative 2 Joint Undertaking under grant agreement No. 115797 (INNODIA). This joint undertaking receives support from the Union’s Horizon 2020 research and innovation program and EFPIA, JDRF, and The Leona M. and Harry B. Helmsley Charitable Trust.

## Conflict of Interest

The authors declare that the research was conducted in the absence of any commercial or financial relationships that could be construed as a potential conflict of interest.

## Publisher’s Note

All claims expressed in this article are solely those of the authors and do not necessarily represent those of their affiliated organizations, or those of the publisher, the editors and the reviewers. Any product that may be evaluated in this article, or claim that may be made by its manufacturer, is not guaranteed or endorsed by the publisher.
