# Supplementary material for: A Humanized Mouse Strain That Develops Spontaneously Immune-Mediated Diabetes
Source: Front Immunol. 2021 Oct 14;12:748679. doi: 10.3389/fimmu.2021.748679 (PMC8551915; doi:10.3389/fimmu.2021.748679)
Supplement: Supplementary file 1 [file DataSheet_1.pdf]

Supplementary Material

1 Supplementary Figures and Tables

1.1 Supplementary Figures

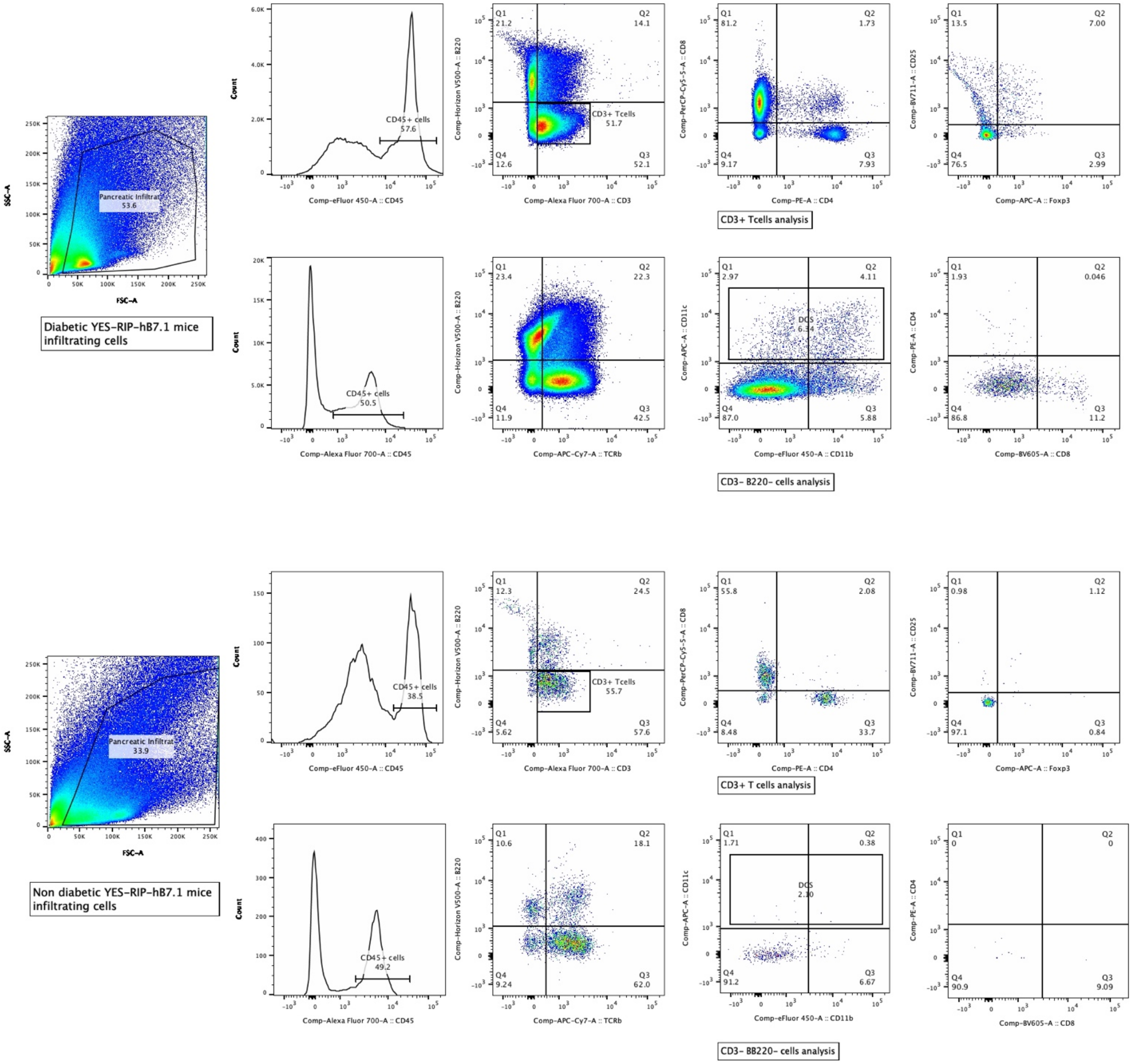

Figure S1. Pancreatic infiltrating cells.

Immune cells recovered from islets of diabetic (upper) or non-diabetic (lower) YES-RIP-hB7.1 mice were analyzed by flow cytometry for T-cells analysis using anti-CD45-efluo450, anti-CD3ε-

AlexaFluo700, anti-B220-HV500, anti-CD8α-Percp-Cy5.5, anti-CD4-PE, anti-Foxp3-APC and anti-CD25-BV711 mAbs. B-cells and for antigen-presenting cells analysis using anti-CD45-AF700, anti-TCRß-APC-Cy7, anti-B220-HV500, anti-CD11b-efluor450, anti-CD11c-APC, anti-CD8α-BV605 and anti-CD4-PE mAbs.

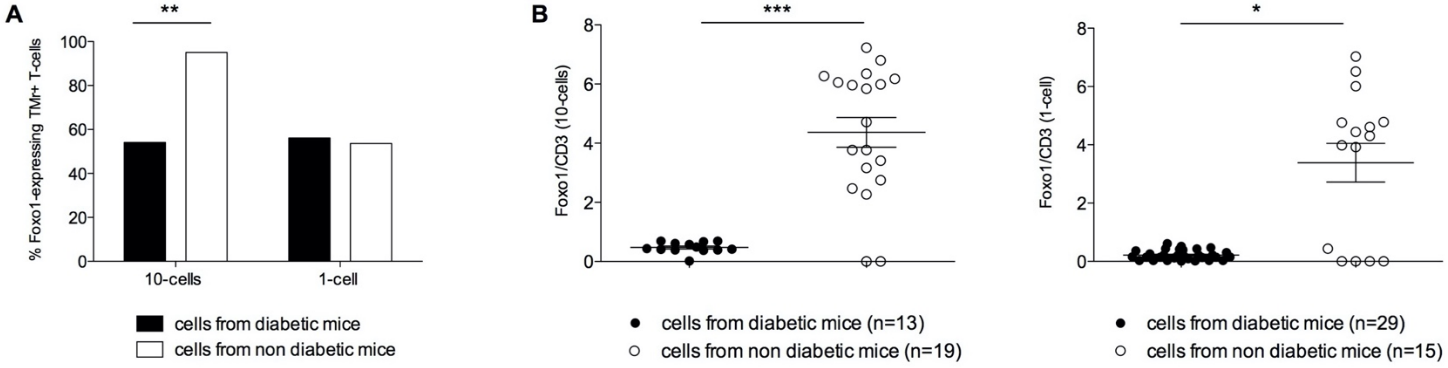

Figure S2. Foxo1-expression in CD8^{+}TMr^{+} T-cells from diabetic and non-diabetic YES-RIP-hB7.1 mice.

(**A**) Multiparametric RT-PCR of hPPI$_{6\text{-}14}$-specific CD8^{+} T-cells. Evaluated-mRNA was *Foxo1* for CD3^{+}CD8^{+}TMr^{+} T-cells from diabetic (closed bars) and non-diabetic (open bars) YES-RIP-hB7.1 mice. Results show the percentage of cells expressing *Foxo1* in diabetic or non-diabetic YES-RIP-hB7.1 mice. (**B**) Semi-quantitative RT-PCR analysis of *Foxo1* and *CD3ε* expression in hPPI$_{6\text{-}14}$-specific CD8^{+} T-cells from diabetic (●) and non-diabetic (○) YES-RIP-hB7.1 mice. Results are expressed relative to the expression of *CD3ε* in the same cells for a pool of 10 cells- (left graph) or for single-cell (right graph).

* $p \leq 0.05$, ** $p \leq 0.01$, *** $p \leq 0.001$; Mann-Whitney test.

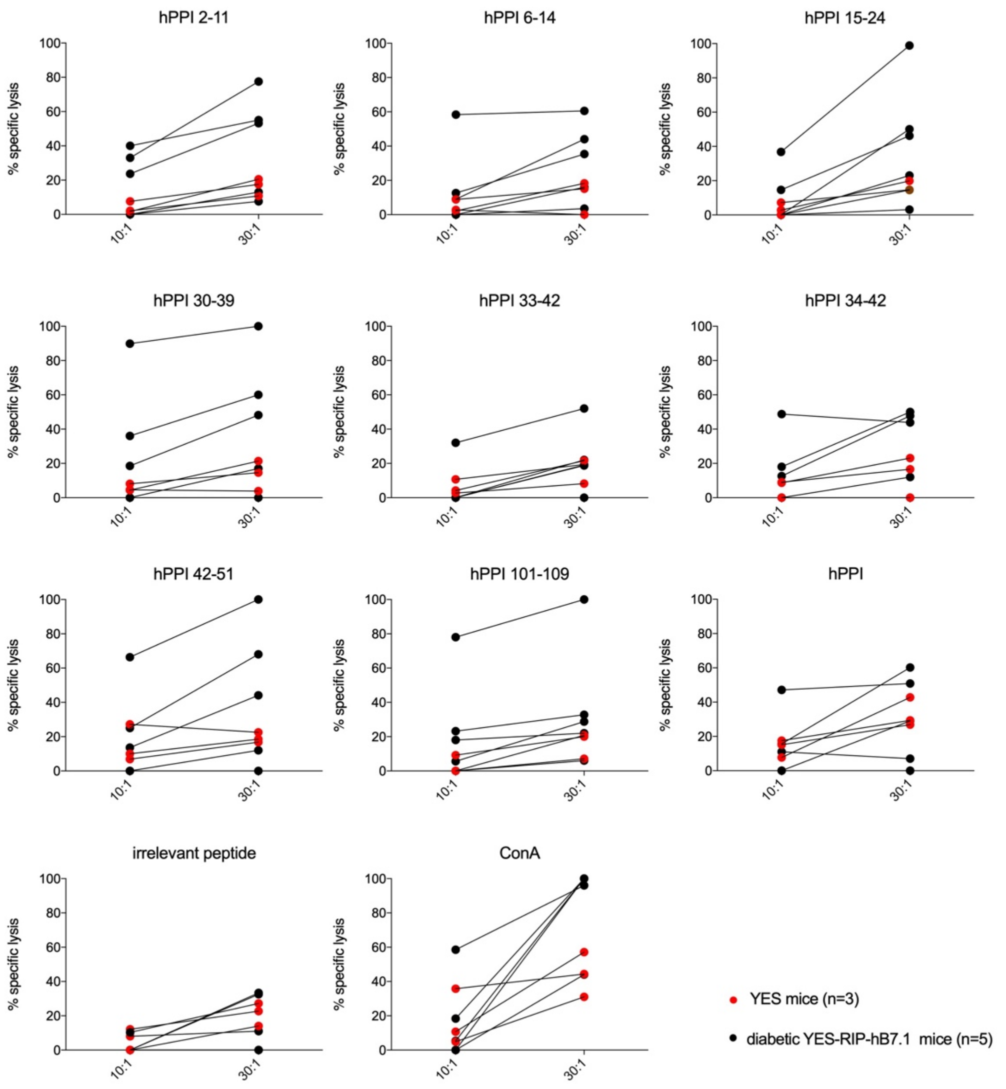

Figure S3. Cytotoxicity of CD8+ T-cells from YES-RIP-hB7.1 against P815 cells transfected with the HHD-encoding gene.

Cytolytic activity of splenocytes from diabetic YES-RIP-hB7.1 (black symbol) and YES control mice (red symbol) were tested against targets loaded with relevant hPPI-peptides or without peptides, measuring of LDH release. The Figure represents the percentage of specific lysis (vertical axis) obtained for 10:0 and 30:1 effector:target ratios (horizontal axis). Cytotoxicity was also tested against P815 cells transfected with the HHD-encoding and the human preproinsulin genes.

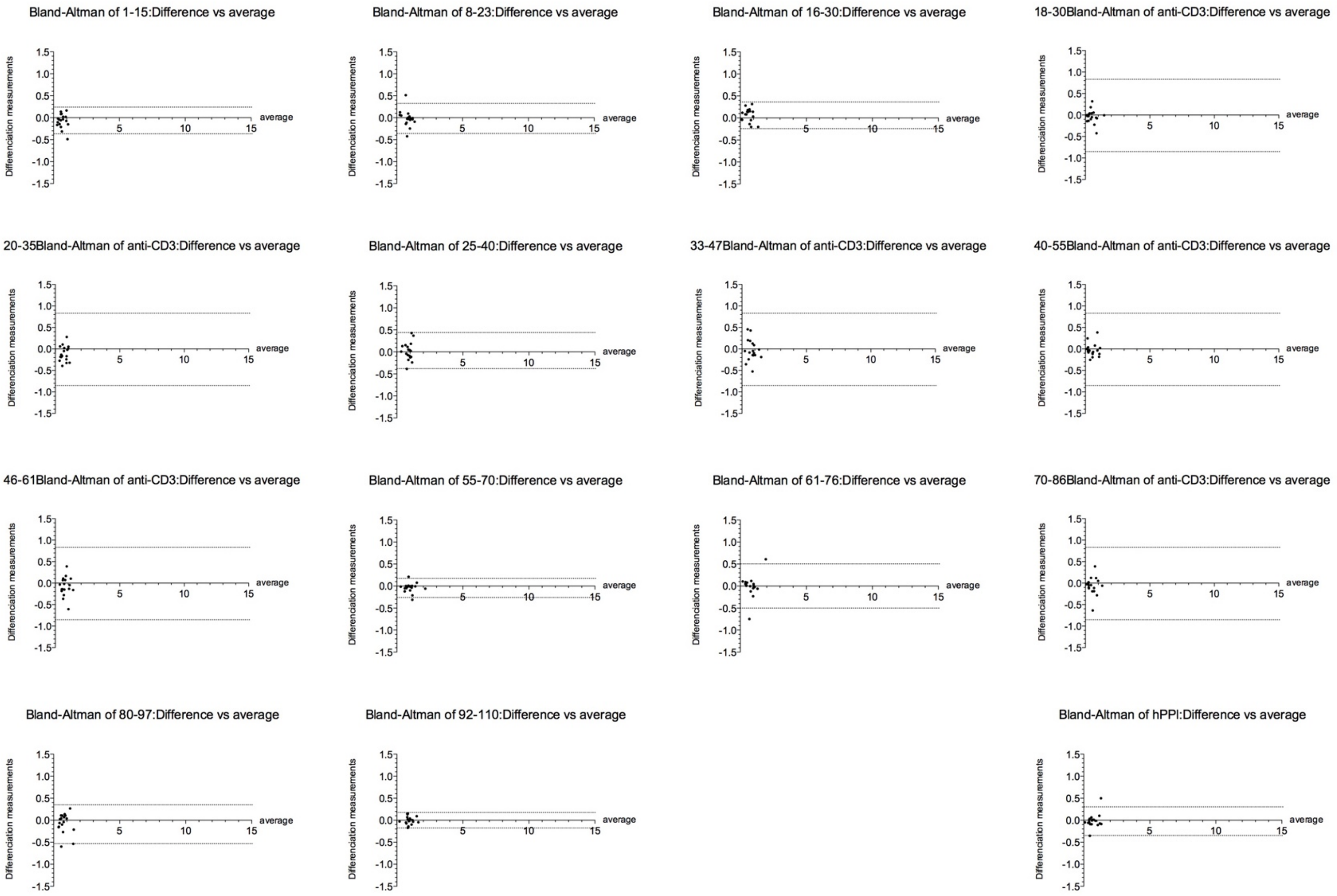

Figure S4. Plots for duplicate measurements score of BrdU-incorporation proliferation assay in YES mice control against hPPI peptides.

The scatter plot of the difference between the measurements against their mean allows detecting no lack of individual reliability which may be hidden by the use of global reliability statistics for significant proliferation responses. The SD of bias emerging from Bland & Altman test allow us to calculate the threshold of a positive response for each peptide in YES mice control.

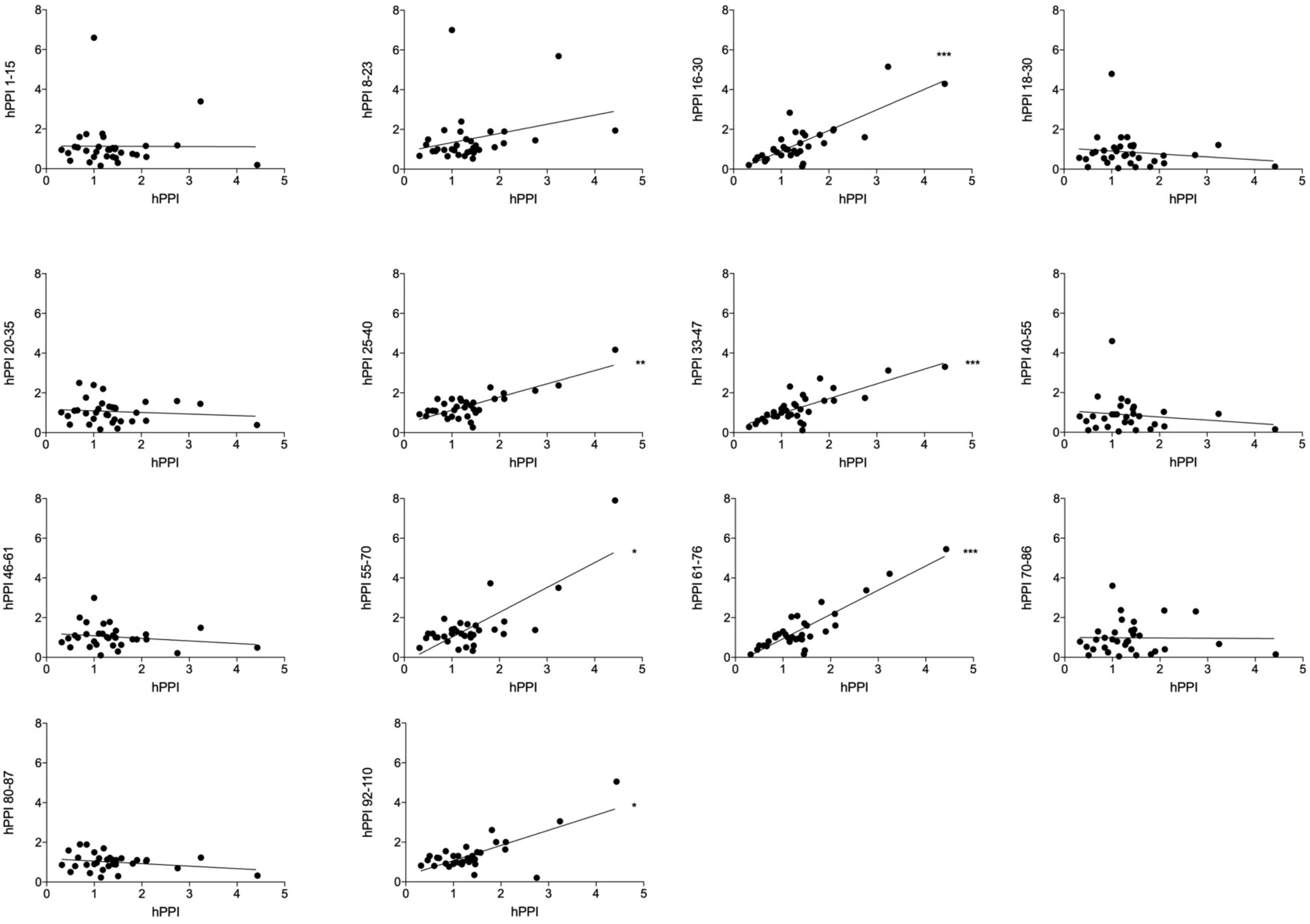

Figure S5. Correlation between hPPI protein and hPPI-peptides proliferative response in diabetic YES-RIP-hB7.1 mice

Proliferation index of hPPI protein were compared to index proliferation of each individually hPPI-peptides by Spearman r correlation analysis.

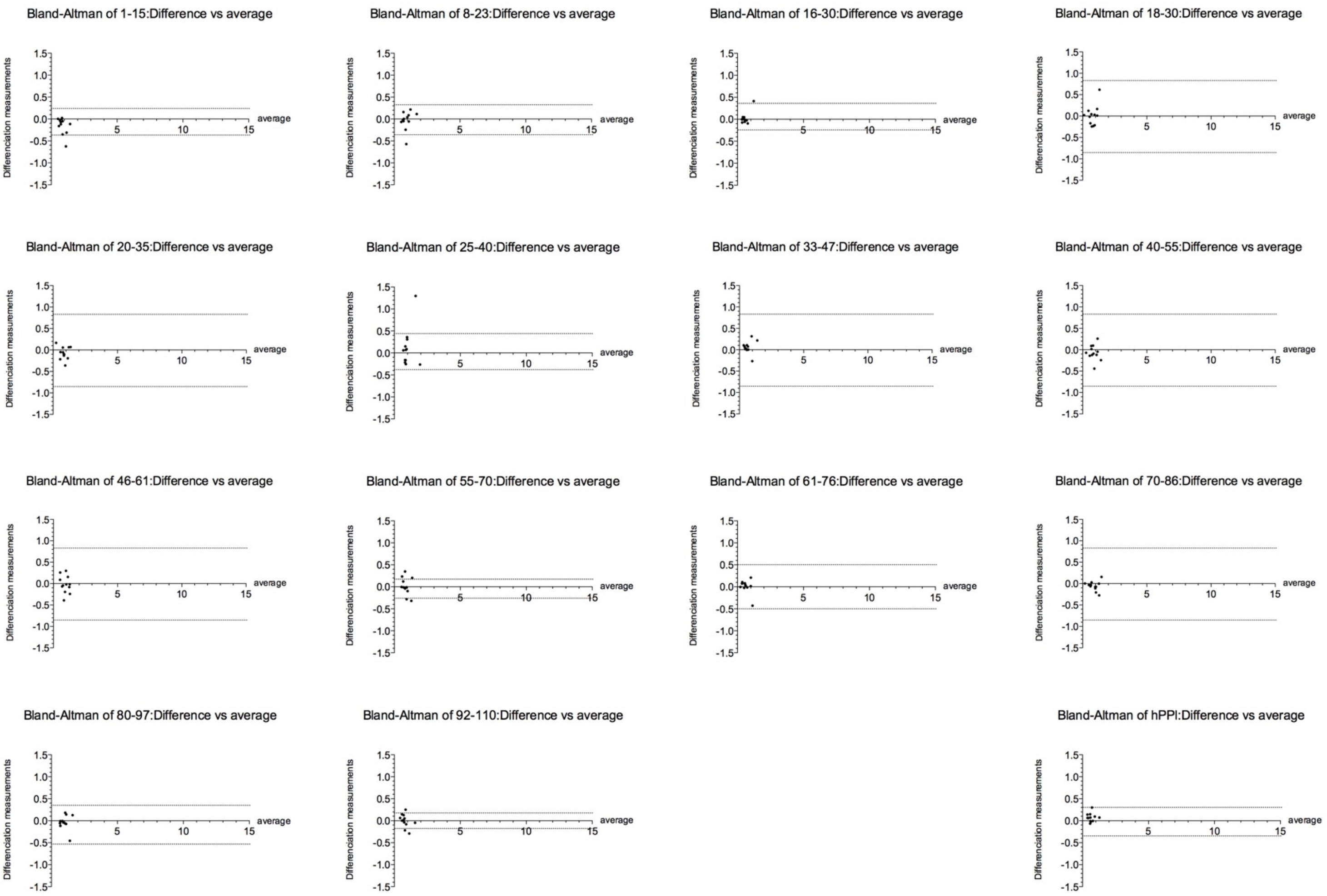

Figure S6. Plots for duplicate measurements score of BrdU-incorporation proliferation assay in HLA-DQ8 human controls against hPPI peptides.

The scatter plot of the difference between the measurements against their mean allows detecting no lack of individual reliability which may be hidden by the use of global reliability statistics for significant proliferation responses. The SD of bias emerging from Bland & Altman test allow us to calculate the threshold of a positive response for each peptide in HLA-DQ8-restricted control (n=12).

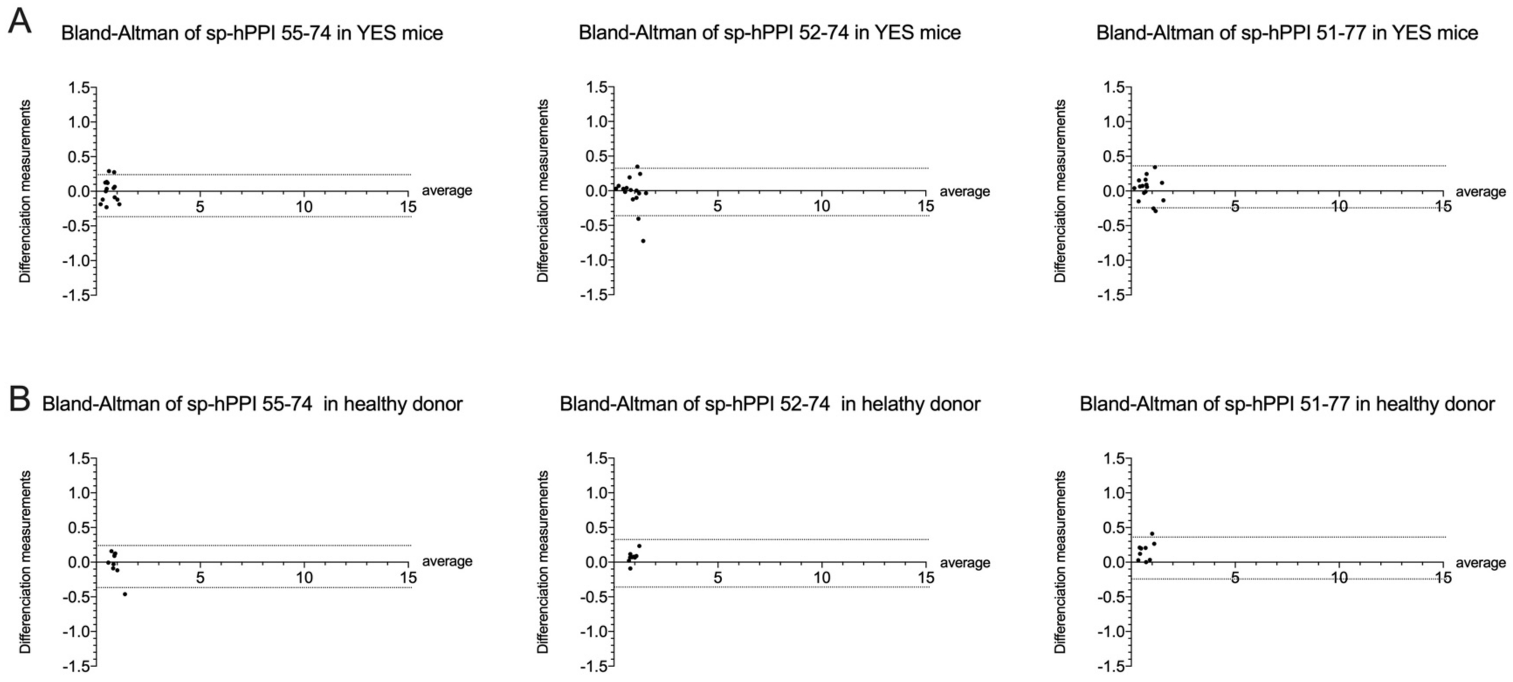

Figure S7. Plots for duplicate measurements score of BrdU-incorporation proliferation assay in YES mouse controls and in HLA-DQ8 human controls against peptides specific to spliced-hPPI.

The scatter plot of the difference between the measurements against their mean allows detecting no lack of individual reliability which may be hidden by the use of global reliability statistics for significant proliferation responses. The SD of bias emerging from Bland & Altman test allow us to calculate the threshold of a positive response for each peptide in *A* YES mice control and in ***B*** healthy donors.

1.2 Supplementary Tables

Table S1. Nucleotidic sequence of primers used for multiparametric PCR.

Target name	Primer name	Primer sequences
ccr7	CCR7-A	CTAGCTGGAGAGAGACAAGA
	CCR7-B	TATCCGTCATGGTCTTGAGC
	CCR7-C	TACGAGTCGGTGTGCTTCAA
cd3e	CD3e-A	ACCAGTGTAGAGTTGACGTG
	CD3e-B	TATGGCTACTGCTGTCAGGT
	CD3e-C	GCTACTACGTCTGCTACACA
gzma	GZMA-A	TCAAATACCATCTGTGCTGG
	GZMA-B	AGAGGGAGCTGACTTATTGC
	GZMA-C	GGGATCTACAACTTGTACGG
gzmb	GZMB-A	GTCAATGTGAAGCCAGGAGA
	GZMB-B	AGGATCCGATGTTGCTTCTG
	GZMB-C	GGGAGTGTGAGTCCTACTTT
ifng	IFNg-A	GCTCTGAGACAATGAACGCT
	IFNg-B	AAAGAGATAATCTGGCTCTGC
	IFNg-C	TGTTTCTGGCTGTTACTGCC
il10ra	IL10RA-A	AACAGTCAGTACTCCAACT
	IL10RA-B	CTGCTCCGTCGTGATAAGTA
	IL10RA-C	CGGCATCATCTATGGGACAA
il2	IL2-A	TCAAGTCCAGCGTAATGT

	IL2-B	CTGTGGTAAGCATGCTCTGT
	IL2-C	CTCTACAGCGGAAGCACAGC
il7r	IL7R-A	GAGTCCAAGTCCTACCTTCG
	IL7R-B	CGGTTTGCACTGTGTACAGC
	IL7R-C	AACCTGTCGTATGGCCTAGT
prf1	PRF1-A	TCACACTGCCAGCGTAATGT
	PRF1-B	CTGTGGTAAGCATGCTCTGT
	PRF1-C	CACAGTAGAGTGTCGCATGT
tgfb	TGFb -A	ACCATCCATGACATGAACCG
	TGFb -B	CAATCATGTTGGACAACTGC
	TGFb -C	GCTACCATGCCAACTTCTGT
tnfsf6	TNFSF6-A	TTCATGGTTCTGGTGGCTCT
	TNFSF6-B	GAGCGGTTCCATATGTGTCT
	TNFSF6-C	TGTATCAGCTCTTCCACCTG
lta	LTA-A	AGCACAGAAAGCATGATCCG
	LTA-B	AACCTGGGAGTAGACAAGGT
	LTA-C	CCTCCCTCTCATCAGTTCTA
tgfbr1	TGFBR1-A	TGTCTCAGTCACTGAGACCA
	TGFBR1-B	AGGTGAATGACAGTGCGGTT
	TGFBR1-C	TGCAATCAGGACCACTGCAA
tgfbr2	TGFBR2-A	AGATGCATCCATCCACCTAA
	TGFBR2-B	TGCACTCTTCCATGTTACAG

	TGFBR2-C	CGATGTGAGACTGTCCACTT
tgfbr3	TGFBR3-A	GAGTGAACGTCCATGCACG
	TGFBR3-B	TGACTGACAGGGCGATATTC
	TGFBR3-C	CATTGGACAATGGCTACAGC
il21	IL21-A	GATCGCCTCCTGATTAGACT
	IL21-B	CTTCGGGTCCTATGTGTTCT
	IL21-C	CACTGTGAGCATGCAGCTTT
il21r	IL21R-A	TCAAGTCCAGCGTAATGT
	IL21R-B	CTGTGGTAAGCATGCTCTGT
	IL21R-C	CTCTACAGCGGAAGCACAGC
1l15	1L15-A	CTTGCAGTGCATCTCCTTAC
	1L15-B	AATGCCCAGGTAAGAGCTTC
	1L15-C	AAGCACTGCCTCTTCATGGT
1l15r	1L15R-A	ATTGAGCATGCTGACATCCG
	1L15R-B	TGTGGTCATTGCGGTATCTG
	1L15R-C	GGTATGTCTGTAACTCTGGC
foxo1	foxo1-A-F	GGACAGCCGCGCAAGACCAG
	foxo1-A-R	ACTGTTGTTGTCCATGGACGC
	foxo1-B-F	ATCACCAAGGCCATCGAGAGC
	foxo1-B-R	TTGAATTCTTCCAGCCCGCCGA

Table S2. *LV-RIP-hB7.1* transgene insertions.

	F36 founder		F36 offspring		Chromosomal position	Closest gene	T1D linkage regions	IDD proximity
Nb initial seq	5 0 831		63 167					
	Begin	End	Begin	End				
% reads mapped	41.11%	40.37%	28.69%	24.58%				
reads mapped	876	1042	683	632				
reads #	1 918		1 315					
Chromosome Region	chr11:75004298-75008247				11qB5	*Smg6*	None	Idd-4.1 (-3Mb) Idd-4.3 (-18Mb)
	chr14:122124274-122128223				14qE5	*Tm9sf2*	None	Idd-8 (-100Mb) Idd-12 (-85Mb)
	chr16:44139732-44142624				16qB4	*Naa50*	None	None
	chr19:15483421-15487432		chr19:15483421-15487432		19qA	None	None	None

# reads obtained after filtering of the captured sequences with the primary LV-RIP-hB7.1 sequence and selection of 50 bp sequences from the beginning or the end of LV-RIP-hB7.1 sequence attached to unknown, presumably mouse sequences.

§ alignment of attached sequences against scaffolds of the C57BL/6NJ lineage using IGV software.

Table S3. IFNγ-ELISpot responses to hPPI peptides in YES-RIP-hB7.1 and YES mice.

Peptide	Responses #			Frequencies of recognition (±3SD)		
	diabetic YES-RIP-hB7.1 mice (n=26)	YES mice (n=14)	*p*value	diabetic YES-RIP-hB7.1 mice	YES mice	*p*value
hPPI $_{2-11}$	148.2(0-917)	48.93 (0-167)	≤0.03	5/26	0/14	
hPPI $_{6-14}$	185.6 (4-462)	63.43 (0-133)	≤0.001	9/26	0/14	≤0.016
hPPI $_{15-24}$	154.2 (0-829)	23.57 (0-100)	≤0.02	11/26	0/14	≤0.004
hPPI $_{30-39}$	93.35 (0-425)	31.93 (0-192)		6/26	1/14	
hPPI $_{33-42}$	179.9 (0-620)	58.43 (0-175)	≤0.013	9/26	0/14	≤0.016
hPPI $_{34-42}$	175.7 (0-812)	55.79 (0-329)		5/26	1/14	
hPPI $_{42-51}$	76.27 (0-325)	39.14 (0-104)		4/26	0/14	
hPPI $_{101-109}$	147.3 (0-1137)	70.64 (0-175)		4/26	0/14	
PDHase $_{208-216}$	64.5 (0-288)	49.78 (0-195)		1/26	0/14	

median (range) of spot number

Table S4. Threshold value determination of BrdU proliferation assay to hPPI peptides in YES mice.

Peptide	YES mice (n=20)	SD of bias	Threshold (mean±3SD)
hPPI $_{1\text{-}15}$	0.62 (0.3-0.95)	0.161	1.11
hPPI $_{8\text{-}23}$	0.87 (0.33-1.3)	0.183	1.42
hPPI $_{16\text{-}30}$	0.71 (0.1-1.2)	0.151	1.17
hPPI $_{18\text{-}30}$	0.55 (0.17-1.3)	0.156	1.02
hPPI $_{20\text{-}35}$	0.8 (0.49-1.25)	0.176	1.33
hPPI $_{25\text{-}40}$	0.96 (0.36-1.4)	0.205	1.57
hPPI $_{33\text{-}47}$	0.83 (0.18-1.5)	0.25	1.58
hPPI $_{40\text{-}55}$	0.61 (0.1-1.26)	0.151	1.06
hPPI $_{46\text{-}61}$	0.85 (0.4-1.4)	0.221	1.51
hPPI $_{55\text{-}70}$	1.01 (0.41-2.3)	0.108	1.33
hPPI $_{61\text{-}76}$	0.86 (0.16-2.2)	0.249	1.61
hPPI $_{70\text{-}86}$	0.59 (0.2-1.28)	0.205	1.20
hPPI $_{80\text{-}97}$	0.79 (0.4-1.6)	0.226	1.47
hPPI $_{92\text{-}110}$	0.98 (0.24-1.8)	0.094	1.26
hPPI [#]	0.77 (0.15-1.5)	0.160	1.25

[#] response against the whole hPPI protein

Table S5. Proliferative responses against hPPI protein and hPPI peptides in YES-RIP-hB7.1 and YES mice.

Peptide	Responses (proliferation index)				Frequencies of recognition (mean±3SD)			
	diabetic YES-RIP-hB7.1 mice (n=33)	YES mice (n=20)	non-diabetic YES-RIP-hB7.1 mice (n=27)	*p*value	diabetic YES-RIP-hB7.1 mice (n=33)	YES mice	non-diabetic YES-RIP-hB7.1 mice	*p*value
hPPI $_{1-15}$	1.13 (0.15-6.6)	0.62 (0.3-0.95)	0.75 (0.24-1.34)	≤0.003	10/33	0/20	3/27	≤0.008
hPPI $_{8-23}$	1.52 (0.53-7)	0.87 (0.33-1.3)	1.91 (0.34-1.9)	≤0.018	12/33	0/20	3/27	≤0.002
hPPI $_{16-30}$	1.34 (0.13-5.1)	0.71 (0.1-1.2)	0.86 (0.18-3.2)	≤0.015	13/33	4/20	4/27	
hPPI $_{18-30}$	0.86 (0.05-4.8)	0.55 (0.17-1.3)	0.76 (0.05-2.2)		10/33	1/20	5/27	≤0.04
hPPI $_{20-35}$	1.06 (0.16-2.5)	0.8 (0.49-1.25)	0.84 (0.05-1.8)		8/33	0/20	3/27	≤0.02
hPPI $_{25-40}$	1.38 (0.26-4.2)	0.96 (0.36-1.4)	0.88 (0.1-1.9)	≤0.014	12/33	0/20	2/27	≤0.002
hPPI $_{33-47}$	2.26 (0.12-3.3)	0.83 (0.18-1.5)	0.83 (0.05-3.37)	≤0.05	10/33	0/20	2/27	≤0.009
hPPI $_{40-55}$	0.87 (0.04-4.6)	0.61 (0.1-1.26)	0.79 (0.08-2.2)		7/33	2/20	3/27	
hPPI $_{46-61}$	1.04 (0.1-3)	0.85 (0.4-1.4)	0.996 (0.23-2.1)		5/33	0/20	2/27	
hPPI $_{55-70}$	1.5 (0.33-7.9)	1.01 (0.41-2.3)	0.99 (0.4-1.7)	≤0.029	13/33	1/20	4/27	≤0.009
hPPI $_{61-76}$	1.42 (0.14-5.4)	0.86 (0.16-2.2)	0.92 (0.2-2.6)	≤0.032	10/33	1/20	3/27	≤0.04
hPPI $_{70-86}$	0.98 (0.05-3.6)	0.59 (0.2-1.28)	0.92 (0.09-3.34)		10/33	1/20	4/27	≤0.04
hPPI $_{80-97}$	1.01 (0.23-1.9)	0.79 (0.4-1.6)	0.95 (0.3-1.8)	≤0.022	5/33	1/20	1/27	
hPPI $_{92-110}$	1.37 (0.2-5.05)	0.98 (0.24-1.8)	0.93 (0.07-1.7)	≤0.028	15/33	1/20	3/27	≤0.002
hPPI #	1.39 (0.32-4.4)	0.77 (0.15-1.5)	0.91 (0.24-2.6)	≤0.0007	17/33	3/20	3/27	≤0.009

response against the whole recombinant hPPI protein

Table S6. Threshold value determination of BrdU proliferation assay in HLA-DQ8 human controls.

Peptide	Control (n=12)	SD of bias	Threshold (mean±3SD)
hPPI $_{1-15}$	0.96 (0.55-1.55)	0.191	1.53
hPPI $_{8-23}$	0.95 (0.51-1.92)	0.208	1.57
hPPI $_{16-30}$	0.6 (0.31-1.23)	0.131	0.99
hPPI $_{18-30}$	0.77 (0.15-1.42)	0.236	1.48
hPPI $_{20-35}$	0.95 (0.24-1.47)	0.147	1,39
hPPI $_{25-40}$	1.03 (0.65-2)	0.431	2.32
hPPI $_{33-47}$	0.71 (0.36-1.38)	0.139	1.13
hPPI $_{40-55}$	0.92 (0.36-1.49)	0.178	1.45
hPPI $_{46-61}$	0.99 (0.52-1.4)	0.201	1.6
hPPI $_{55-70}$	0.93 (0.58-1.42)	0.196	1.52
hPPI $_{61-76}$	0.65 (0.22-1.27)	0.192	1.23
hPPI $_{70-86}$	0.89 (0.21-1.47)	0.11	1.22
hPPI $_{80-97}$	1.04 (0.54-1.7)	0.162	1.53
hPPI $_{92-110}$	0.88 (0.58-1.6)	0.151	1.33
hPPI [#]	0.65 (0.33-1.33)	0.069	0.86

[#] response against the whole recombinant hPPI protein

Table S7. Proliferative responses against hPPI and hPPI peptides in T1D HLA-DQ8 patients.

Peptide	Responses (Proliferation Index)			Frequencies of recognition (mean±3SD)		
	Recent-onset T1D patients (n=11)	Controls (n=13)	*pvalue*	T1D patients (n=11)	Controls (n=13)	*pvalue*
hPPI $_{1\text{-}15}$	2.1 (0.69-4.5)	1.03 (0.55-1.98)	≤0.007	8/11	2/13	≤0.01
hPPI $_{8\text{-}23}$	1.57 (0.78-2.64)	1.04 (0.51-2.05)	≤0.003	4/11	2/13	
hPPI $_{16\text{-}30}$	0.93 (0.2-2.76)	0.65 (0.31-1.25)		4/11	2/13	
hPPI $_{18\text{-}30}$	1.73 (0.49-4.44)	0.81 (0.15-1.42)	≤0.016	5/11	0/13	≤0.01
hPPI $_{20\text{-}35}$	2.14 (0.88-4.15)	1.01 (0.24-1.65)	≤0.0007	8/11	3/13	≤0.04
hPPI $_{25\text{-}40}$	1.53 (0.58-2.32)	1.07 (0.65-2)	≤0.027	1/11	0/13	
hPPI $_{33\text{-}47}$	1.01 (0.22-2.19)	0.73 (0.36-1.38)		5/11	0/13	
hPPI $_{40\text{-}55}$	1.81 (0.48-4.39)	0.96 (0.36-1.49)		6/11	1/13	≤0.003
hPPI $_{46\text{-}61}$	1.82 (0.68-3.45)	1.05 (0.52-1.79)	≤0.03	6/11	1/13	≤0.02
hPPI $_{55\text{-}70}$	1.21 (0.5-2.04)	0.96 (0.58-1.42)		3/11	0/13	
hPPI $_{61\text{-}76}$	0.84 (0.14-1.54)	0.66 (0.22-1.27)		2/11	1/13	
hPPI $_{70\text{-}86}$	1.78 (0.4-5.54)	0.9 (0.21-1.47)	≤0.045	7/11	3/13	
hPPI $_{80\text{-}97}$	1.87 (0.87-3.58)	1.08 (0.54-1.7)	≤0.018	7/11	2/13	≤0.03
hPPI $_{92\text{-}110}$	1.37 (0.28-5.37)	0.1 (0.58-1.6)	≤0.03	5/11	1/13	
hPPI $^{\#}$	1.85 (0.28-5.37)	0.69 (0.33-1.33)	≤0.04	8/11	3/13	≤0.04

$^{\#}$ response against the whole recombinant hPPI protein

Table S8. Proliferative responses against hPPI and hPPI peptides in T1D HLA-DQ2/DQ8 patients

Peptide	Responses (Proliferation Index)			Frequencies of recognition (mean±3SD)		
	Recent-onset T1D patients (n=19)	Controls (n=13)	*pvalue*	T1D patients (n=19)	Controls (n=13)	*pvalue*
hPPI $_{1\text{-}15}$	1.68 (0.45-5.5)	1.03 (0.55-1.98)		7/19	2/13	
hPPI $_{8\text{-}23}$	1.8 (0.52-4.97)	1.04 (0.51-2.05)	≤0.009	10/19	2/13	
hPPI $_{16\text{-}30}$	1.4 (0.13-5.76)	0.65 (0.31-1.25)		10/19	2/13	
hPPI $_{18\text{-}30}$	1.23 (0.13-5.06)	0.81 (0.15-1.42)		4/19	0/13	
hPPI $_{20\text{-}35}$	1.6 (0.77-5.61)	1.01 (0.24-1.65)	≤0.05	7/19	3/13	
hPPI $_{25\text{-}40}$	1.87 (0.41-7.44)	1.07 (0.65-2)	≤0.05	4/19	0/13	
hPPI $_{33\text{-}47}$	1.26 (0.05-5.16)	0.73 (0.36-1.38)		8/19	0/13	≤0.05
hPPI $_{40\text{-}55}$	1.39 (0.11-5.08)	0.96 (0.36-1.49)		7/19	1/13	≤0.03
hPPI $_{46\text{-}61}$	1.68 (0.82-6.38)	1.05 (0.52-1.79)		5/19	1/13	
hPPI $_{55\text{-}70}$	1.7 (0.4-4.64)	0.96 (0.58-1.42)	≤0.003	11/19	0/13	≤0.0006
hPPI $_{61\text{-}76}$	1.25 (0.06-4.34)	0.66 (0.22-1.27)		6/19	1/13	
hPPI $_{70\text{-}86}$	1.23 (0.12-4.84)	0.9 (0.21-1.47)		7/19	3/13	
hPPI $_{80\text{-}97}$	1.57 (0.7-5.53)	1.08 (0.54-1.7)		5/19	2/13	
hPPI $_{92\text{-}110}$	1.6 (0.43-4.13)	0.1 (0.58-1.6)	≤0.04	9/19	1/13	≤0.03
hPPI #	1.99 (0.13-6.31)	0.69 (0.33-1.33)	≤0.003	14/19	3/13	≤0.001

response against the whole recombinant hPPI protein

Table S9. Threshold value determination of BrdU proliferation against spliced-hPPI peptides in YES mice.

Peptide	YES mice (n=16)	SD of bias	Threshold (mean±3SD)
sp-hPPI$_{55\text{-}74}$	0.77 (0.21-2.66)	0.443	2.01
sp-hPPI$_{52\text{-}74}$	0.89 (0.105-1.54)	0.249	1.64
sp-hPPI$_{51\text{-}77}$	0.78 (0.146-1.545)	0.171	1.29

Table S10. Threshold value determination of BrdU proliferation assay in HLA-DQ8 human controls.

Peptide	Control (n=9)	SD of bias	Threshold (mean±3SD)
sp-hPPI$_{55\text{-}74}$	0.77 (0.21-2.63)	0.191	1.34
sp-hPPI$_{52\text{-}74}$	0.89 (0.1-1.54)	0.085	1.14
sp-hPPI$_{51\text{-}77}$	0.78 (0.15-1.54)	0.132	1.18

Table S11. Proliferative responses against spliced hPPI peptides in YES-RIP-hB7.1 and YES mice.

Peptide	Responses (proliferation index)				Frequencies of recognition (mean±3SD)	
	diabetic YES-RIP-hB7.1 mice (n=40)	YES mice (n=22)	non-diabetic YES-RIP-hB7.1 mice (n=15)	*p*value	diabetic YES-RIP-hB7.1 mice	YES mice
sp-hPPI$_{55\text{-}74}$	1.31 (0.3-5.1)	0.95 (0.3-2.7)	0.63 (0.24-1.34)		6/40	2/22
sp-hPPI$_{52\text{-}74}$	1.57 (0.4-5.2)	0.96 (0-2)	10.91 (0.16-3.5)	≤0.0086	12/40	3/22
sp-hPPI$_{51\text{-}77}$	1.19 (0.16-3.49)	0.83 (0.12-1.6)	0.69 (0.2-1.3)	≤0.049	13/40	2/22
